# A role for the transcriptional coregulator RIP140 in the control of muscle endurance fitness

**DOI:** 10.1172/jci.insight.192376

**Published:** 2025-10-21

**Authors:** Elizabeth Pruzinsky, Kirill Batmanov, Denis M. Medeiros, Sarah M. Sulon, Brian P. Sullivan, Tomoya Sakamoto, Teresa C. Leone, Tejvir S. Khurana, Daniel P. Kelly

**Affiliations:** 1 Cardiovascular Institute, Perelman School of Medicine, University of Pennsylvania, Philadelphia, Pennsylvania, USA.; 2 Division of Biological and Biomedical Systems, School of Science and Engineering, The University of Missouri-Kansas City, Kansas City, Missouri, USA.; 3 Pennsylvania Muscle Institute, Department of Physiology, Perelman School of Medicine, University of Pennsylvania, Philadelphia, Pennsylvania, USA.

**Keywords:** Metabolism, Muscle biology, Mitochondria, Skeletal muscle, Transcription

## Abstract

Poor skeletal muscle fitness contributes to many chronic disease states, including obesity, heart failure, primary muscle disorders, and age-related sarcopenia. Receptor-interacting protein 140 (RIP140) is a striated muscle–enriched nuclear receptor coregulator known to suppress mitochondrial oxidative capacity. To investigate the role of RIP140 in skeletal muscle, striated muscle–specific RIP140-deficient (str*Nrip1^–/–^*) mice were generated and characterized. str*Nrip1^–/–^* mice displayed an enhanced endurance performance phenotype. RNA-sequence (RNA-seq) analysis of glycolytic fast-twitch muscle from str*Nrip1^–/–^* mice identified a broad array of differentially upregulated metabolic and structural muscle genes known to be induced by endurance training, including pathways involved in mitochondrial biogenesis and respiration, fatty acid oxidation, slow muscle fiber type, and angiogenesis. In addition, muscle RIP140 deficiency induced expansive neuromuscular junction (NMJ) remodeling. Integration of RNA-seq results with CUT&RUN analysis of str*Nrip1^–/–^* myotubes identified Wnt16 as a candidate effector for the NMJ biogenesis in RIP140-deficient skeletal myotubes. We conclude that RIP140 serves as a physiological “rheostat” for a broad coordinated network of metabolic and structural genes involved in skeletal muscle fitness.

## Abstract 2

Daniel Kelly and team show that the protein RIP140 serves a key role in modulating the transcriptional control of skeletal muscle endurance fitness and the exercise training response.

## Graphical abstract

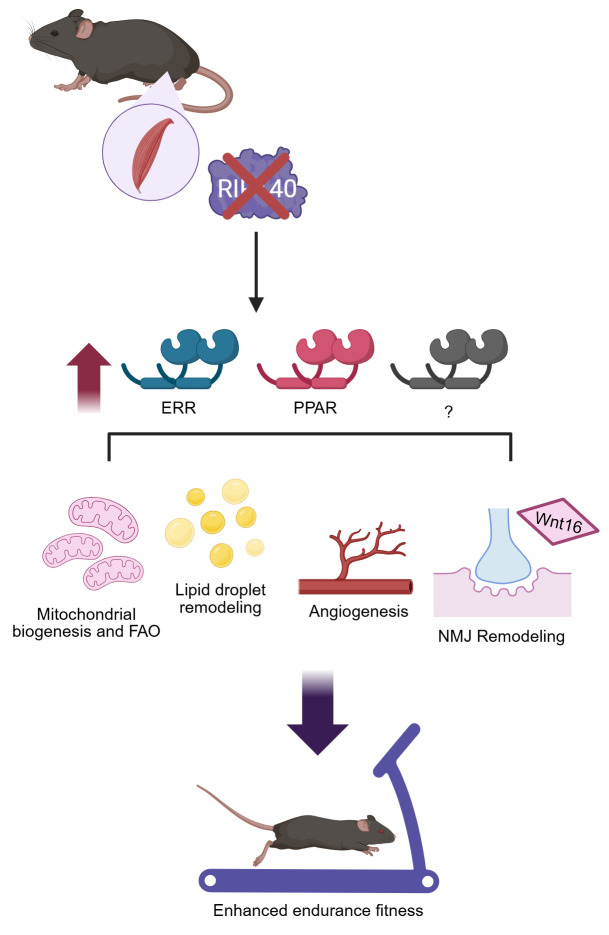

## Introduction

Poor muscle fitness, largely as a result of physical inactivity, occurs with many chronic diseases including obesity, cancer, heart failure, and age-related sarcopenia states. Enhancing muscle functional fitness through regular aerobic exercise training regimens in patients with chronic disease has been shown to improve quality of life, functionality, and health outcomes (1–4). Delineation of the molecular regulatory mechanisms involved in exercise-induced skeletal muscle fitness could lead to new strategies aimed at enhancing and maintaining optimal muscle function in the context of aging and chronic diseases.

Regular aerobic exercise has been shown to elicit an array of stereotypic physiologic, metabolic, and structural adaptive changes in skeletal muscle, including enhanced endurance performance, increased mitochondrial functional capacity, a shift toward oxidative fibers, and angiogenesis (5). The mechanisms involved in the integrative control of these processes with regular physical activity are incompletely understood. Previous studies have shown an important role for the nuclear receptors, the peroxisome proliferator-activated receptor (PPAR) and the estrogen-related receptor (ERR), in transducing exercise to the transcriptional control of muscle fuel metabolism, mitochondrial function, and the oxidative fiber program (6, 7). We and others have shown that transgenic overexpression of PPARδ (8–10) and ERRα and -γ (11–15) in mice results in an impressive endurance phenotype with evidence of increased capacity for fatty acid oxidation, and a shift toward oxidative fiber types, key signatures of endurance fitness. In addition, muscle overexpression of the transcriptional coregulator PPARγ coactivator-1α (PGC-1α), an exercise-induced coactivator of ERR and PPAR (16), also results in an endurance phenotype (17, 18).

The impressive phenotypes of the PPARδ, ERRα/γ, and PGC-1α transgenic mouse lines have provided important insight into transcriptional drivers of muscle metabolic fitness. However, these studies relied on overexpression techniques that are supraphysiologic and difficult to translate into therapeutic strategies. We hypothesized that endogenous coregulators of the PGC-1/nuclear receptor axis modulate this circuitry. If so, targeting such regulators may prove to be a means of enhancing the integrated endurance response within a physiological range, in contrast with the supraphysiological effects of transcription factor overexpression. To this end, we focused on the nuclear receptor coregulator, receptor-interacting protein 140 (RIP140), encoded by *Nrip1*. RIP140 has been shown to act as a corepressor for PPAR and ERR in adipose and other cell types (19–24). Generalized RIP140-null mice display resistance to diet-induced obesity related to enhanced mitochondrial oxidative capacity and uncoupling in brown and white adipose tissue (20, 21). Notably, skeletal muscle of generalized RIP140-null mice exhibit an increased proportion of oxidative fibers (19). However, the direct effect of RIP140 deficiency on muscle metabolism and structure has not been defined. Herein, we demonstrate that mice with muscle-specific RIP140 deficiency exhibit an endurance phenotype that is further enhanced by exercise training. Genomic and phenotypic interrogation of the RIP140-deficient muscle demonstrated activation of a wide array of metabolic and structural endurance fitness signatures, including the surprising finding of neuromuscular junction (NMJ) expansion. Transcriptional enhancer profiling of skeletal myocytes isolated from striated muscle–specific RIP140-deficient (str*Nrip1^–/–^*) mice using H3K27ac cleavage under target and release using nuclease (CUT&RUN) analysis identified Wnt16 as a potential regulator of NMJ biogenesis downstream of the suppressive effect of RIP140.

## Results

### strNrip1^–/–^ mice display an oxidative muscle phenotype that is prominent in fast-twitch muscle.

**Figure 1:**
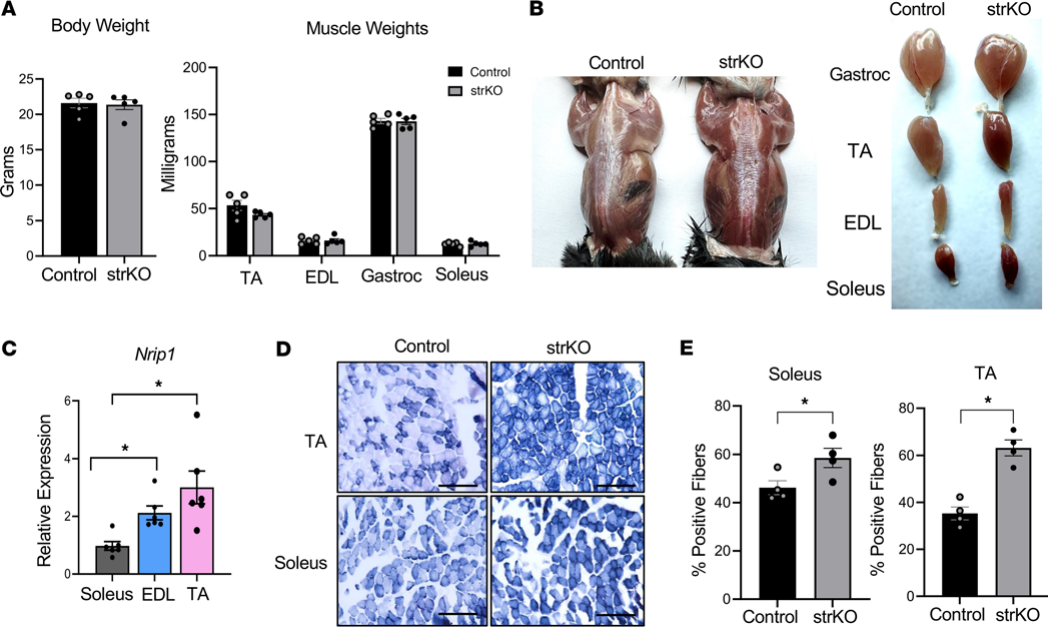
Striated muscle–specific RIP140-KO (str*Nrip1^–/–^*) mice display an oxidative muscle phenotype that is prominent in fast-twitch muscles. (**A**) Body and muscle weights comparing 8-week-old WT control and str*Nrip1*^–/–^ (strKO) male mice (*n* = 5 mice per group). (**B**) Photographs of whole body and individual muscles of 8-week-old WT control and strKO male mouse muscle. Gastrocnemius (gastroc), tibialis anterior (TA), extensor digitalis longus (EDL), and soleus. (**C**) *Nrip1* gene expression in soleus (slow-twitch), EDL (fast-twitch), and TA (fast-twitch) muscle in 8-week-old WT control male mice (*n* = 6 mice per group). (**D**) Representative images of succinate dehydrogenase (SDH) staining of WT control and strKO TA and soleus muscle (*n* = 4 animals per group per muscle type). Scale bars: 50 μm. (**E**) Quantification of SDH-positive fibers in soleus and TA muscle comparing control and strKO male mice (*n* = 4 animals per group). Values shown are mean ± SEM. *P < 0.05 versus control by 2-tailed, unpaired Student’s *t* test (**A**, **C**, and **E**).

str*Nrip1^–/–^* mice were generated as we have previously described using a muscle creatine kinase (MCK) promoter–driven Cre recombinase that targets heart and skeletal muscle (25). The str*Nrip1^–/–^* mice have similar body and muscle weights compared to age-matched littermate WT controls (Figure 1A). The gross appearance of the str*Nrip1^–/–^* muscle was significantly darker and red in color compared with WT controls (Figure 1B). This difference was most apparent in fast-twitch muscles such as extensor digitalis longus (EDL), tibialis anterior (TA), and gastrocnemius compared with the more oxidative fiber–enriched soleus (Figure 1B), consistent with the greater expression of *Nrip1* in glycolytic, fast-twitch muscle (Figure 1C). Corroborating these results, available single-nucleus RNA-sequencing (RNA-seq) data showed higher *Nrip1* expression in type 2B myonuclei in TA muscle and low expression in type 1 and 2A myonuclei in soleus muscle (26). These results are indicative of a shift toward more oxidative fibers, particularly in fast-twitch muscle. This conclusion was further supported by the observation that succinate dehydrogenase (SDH) staining, a measure of mitochondrial oxidative capacity, is increased in str*Nrip1^–/–^* muscle (Figure 1, D and E). The increased SDH staining was more evident in the str*Nrip1^–/–^* TA compared with soleus, further indicating a greater impact of *Nrip1* KO in fast-twitch compared with slow-twitch muscle (Figure 1, D and E).

### Deficiency of RIP140 in skeletal muscle results in enhanced endurance performance.

**Figure 2:**
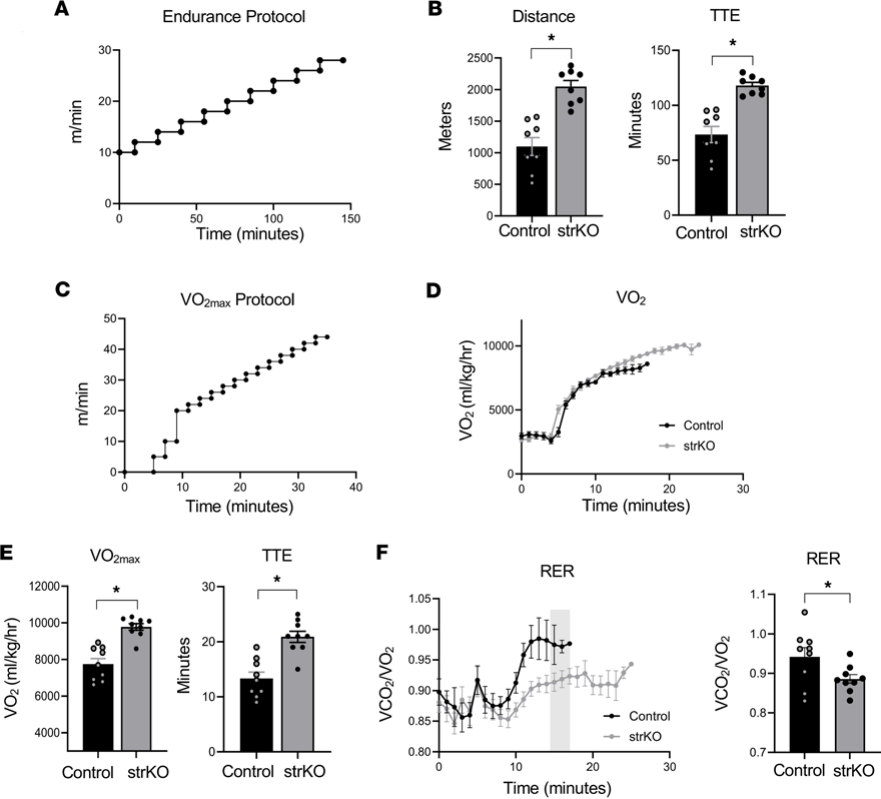
str*Nrip1*^–/–^ mice display enhanced endurance performance. (**A**) Schematic of the motorized treadmill endurance performance protocol. (**B**) Endurance performance results showing distance and time to exhaustion (TTE) of 8-week-old WT control and str*Nrip1*^–/–^ (strKO) male mice (*n* = 8 mice per group). (**C**) Schematic of the motorized treadmill VO_2max_ protocol. (**D**) VO_2_ of 8-week-old male WT control and strKO mice plotted against time using the VO_2max_ exercise protocol (*n* = 9 mice per group). (**E**) VO_2max_ and TTE comparing WT control and strKO mice from the VO_2max_ exercise protocol. (**F**) Respiratory exchange ratio (RER) of WT control and strKO mice throughout the VO_2max_ protocol. The averaged point of WT control exhaustion is shown as the shaded region in light gray and shown as a comparative plot (*n* = 9 mice per group). Values represent mean ± SEM. *P < 0.05 versus control by 2-tailed, unpaired Student’s *t* test (**B**, **E**, and **F**).

The exercise performance of str*Nrip1^–/–^* and WT controls was assessed utilizing an endurance protocol on a motorized treadmill (Figure 2A). Approximately 8% of mice were deemed non-runners in this mouse line. Male str*Nrip1^–/–^* mice displayed an enhanced endurance performance, indicated by a significant increase in distance and time to exhaustion (Figure 2B). To further investigate this phenotype, the maximum rate of oxygen consumption (VO_2max_) was assessed during exercise to exhaustion as shown in Figure 2C. Male str*Nrip1^–/–^* mice exhibited a markedly greater VO_2max_ compared with WT controls, as well as increased time to exhaustion (Figure 2, D and E). The respiratory exchange ratio (RER) was determined during the VO_2max_ protocol as a general measure of fuel utilization. Compared with WT controls, RER was maintained at a significantly lower level in str*Nrip1^–/–^* mice during the exercise period indicative of greater reliance on fatty acid oxidation during exercise, consistent with a trained phenotype (Figure 2F). This was confirmed by calculating the percentage of carbohydrate and fat oxidation throughout the VO_2max_ exercise protocol, indicating str*Nrip1^–/–^* mice utilize fatty acids as fuel to a greater degree compared with WT control mice (Supplemental Figure 1, A and B; supplemental material available online with this article; https://doi.org/10.1172/jci.insight.192376DS1).

Female str*Nrip1^–/–^* mice displayed a similar exercise phenotype to that observed in the male str*Nrip1^–/–^* mice (Supplemental Figure 2, A–E); however, the effect of RIP140 depletion was less pronounced due to enhanced endurance performance in WT control female mice at baseline compared with male mice likely due to greater baseline fatty acid oxidation capacity, as described previously (27, 28). Concordantly, *Nrip1* expression levels were lower in WT control female mice compared with males (Supplemental Figure 2F).

Given that RIP140 was depleted in both heart and skeletal muscle in the str*Nrip1^–/–^* mice, we sought to determine the relative contribution of RIP140 deficiency in heart and skeletal muscle to the endurance phenotype of str*Nrip1^–/–^* mice. As a first step, ex vivo muscle fatiguability and force generation studies were performed in fast-twitch (EDL) and slow-twitch (soleus) muscle isolated from male str*Nrip1^–/–^* and WT control mice. str*Nrip1^–/–^* EDL displayed decreased fatiguability compared with WT control EDL, while soleus showed no significant change (Supplemental Figure 3, A and B). This result is concordant with the greater effect of RIP140 depletion on fast-twitch skeletal muscle. Interestingly, recovery of force generation following completion of the fatigue protocol was also improved in str*Nrip1^–/–^* EDL compared with WT control EDL (Supplemental Figure 3C). No significant change was seen in maximum tetanic force in either EDL or soleus between str*Nrip1^–/–^* and WT control mice (Supplemental Figure 3D). These results indicate a skeletal muscle–autonomous contribution to the endurance phenotype observed in the str*Nrip1^–/–^* mouse.

To further evaluate the contribution of muscle RIP140 depletion to the endurance phenotype of str*Nrip1^–/–^* mice, skeletal muscle–specific RIP140-deficient mice (skm*Nrip1^–/–^* mice) were generated using a myogenin-Cre recombinase driver (29) (Supplemental Figure 4, A and B). The general phenotype of skm*Nrip1^–/–^* mice phenocopied that of the str*Nrip1^–/–^* mice, including normal growth, muscle weight, and appearance (Supplemental Figure 4, C and D). Approximately 11% of mice were deemed non-runners in this mouse line. Notably, skm*Nrip1^–/–^* mice exhibit enhanced endurance performance like that of str*Nrip1^–/–^* mice, including an increase in distance and time to exhaustion (Supplemental Figure 5A), a significantly greater VO_2max_, and decreased RER compared with WT controls (Supplemental Figure 5, B–D). Additionally, skm*Nrip1^–/–^* mice display greater utilization of fatty acids and subsequently less carbohydrate utilization during exercise compared with WT control mice (Supplemental Figure 5, E and F). Directly comparing VO_2max_ levels, time to exhaustion, and RER from str*Nrip1^–/–^* and skm*Nrip1^–/–^* mice showed no difference between these models (Supplemental Figure 6, A–E). Taken together, these findings indicate that depletion of RIP140 in skeletal muscle drives the enhanced endurance phenotype of str*Nrip1^–/–^* mice.

### Augmentation of the endurance phenotype of strNrip1^–/–^ mice by exercise training.

**Figure 3:**
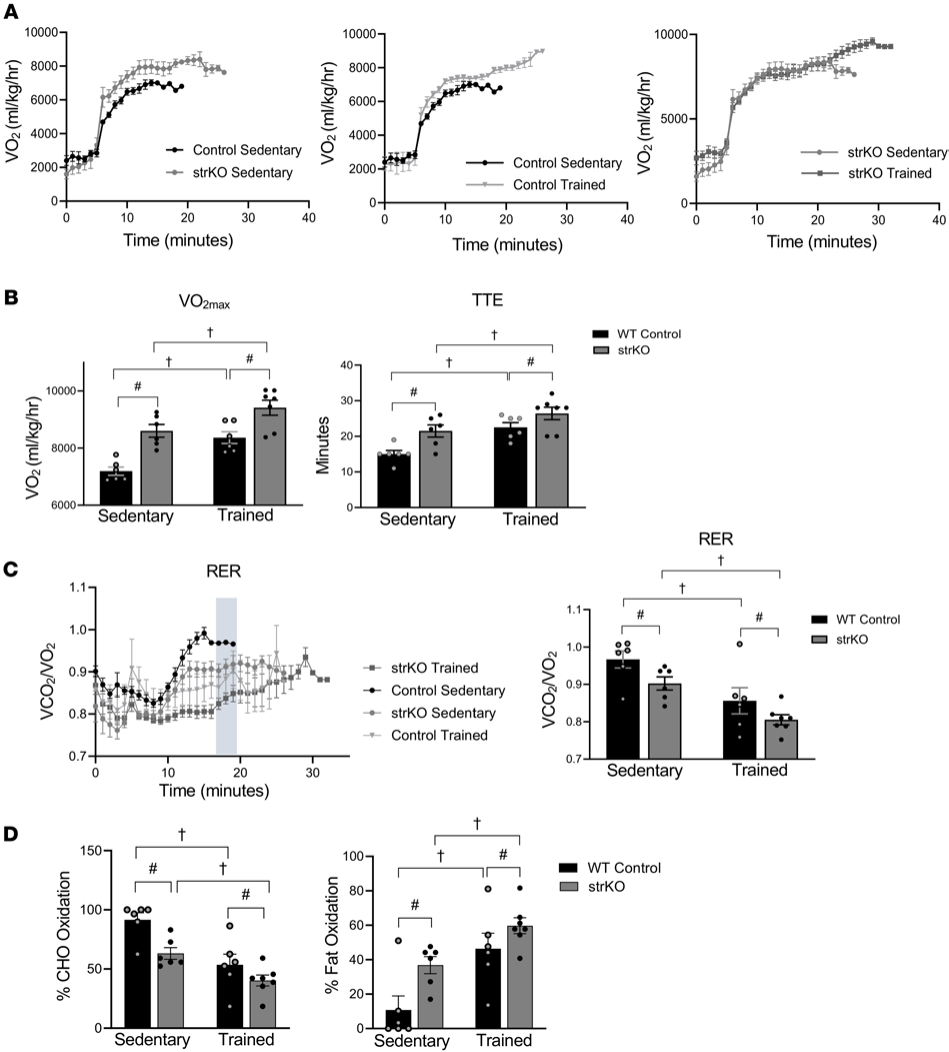
str*Nrip1*^–/–^ mice are capable of training to a VO_2max_ beyond baseline endurance phenotype. (**A**) VO_2_ plotted against time of the VO_2max_ exercise protocol comparing WT control sedentary and str*Nrip1*^–/–^ (strKO) sedentary mice, WT control sedentary and trained WT control mice, and strKO sedentary and strKO trained mice (*n* = 6–7 mice per group). (**B**) VO_2max_ and time-to-exhaustion (TTE) values from the VO_2max_ exercise protocol comparing WT control sedentary versus trained, and strKO sedentary versus trained male mice (*n* = 6–7 mice per group). (**C**) Respiratory exchange ratio (RER) derived from the VO_2max_ exercise protocol comparing WT control sedentary, WT control trained, strKO sedentary, and strKO trained male mice at 16 weeks of age and plotted at the averaged point of WT control exhaustion shaded in gray (*n* = 6–7 mice per group). (**D**) Percentage carbohydrate (CHO) and fat oxidation calculated from RER measurements derived from the VO_2max_ testing protocol comparing WT control sedentary, WT control trained, strKO sedentary, and strKO trained male mice (*n* = 6–7 mice per group). Values are the mean ± SEM. ^#^*P* < 0.05 comparing genotypes and ^†^*P* < 0.05 comparing training by 2-way ANOVA displaying main effects (**B**–**D**).

Given the impressive endurance performance of the str*Nrip1^–/–^* mice, we next determined whether endurance training could further enhance this phenotype. To this end, 8-week-old male str*Nrip1^–/–^* and WT control littermate mice were subjected to either nocturnal voluntary wheel running or sedentary conditions for 8 weeks. str*Nrip1^–/–^* and WT control littermate mice ran similar distances per night (Supplemental Figure 7A). Body composition measurements did not display significant differences between any groups (Supplemental Figure 7B). At the completion of the training period, the 4 groups (sedentary WT control, sedentary str*Nrip1^–/–^*, WT control trained, and str*Nrip1^–/–^* trained) underwent VO_2max_ testing. The data revealed significant effects of training (*P* = 0.0002) and genotype (*P* < 0.0001) on VO_2max_ and time to exhaustion, with no significant training × genotype interaction (Figure 3, A and B). Thus, training increased endurance similarly in both WT and str*Nrip1^–/–^* mice, and str*Nrip1^–/–^* animals exhibited higher values overall. Similar results were observed in distance and time to exhaustion in treadmill endurance testing (Supplemental Figure 7C). A comparable pattern was observed for RER (Figure 3C; training effect, *P* = 0.0002; genotype effect, *P* = 0.02; no interaction). Consistent with the RER results, the calculated percentage of fatty acid oxidation during the exercise protocol was further increased by training in WT and str*Nrip1^–/–^* mice (Figure 3D). Concordantly, the percentage of carbohydrate oxidation was further decreased by training in both genotypes (Figure 3D). Notably, *Nrip1* expression was not significantly different between WT control trained and sedentary muscle (Supplemental Figure 7D). Altogether, these data indicate that the enhanced endurance phenotype of str*Nrip1^–/–^* mice can be further improved by exercise.

### Muscle RIP140 deficiency activates a broad muscle endurance fitness network.

**Figure 4:**
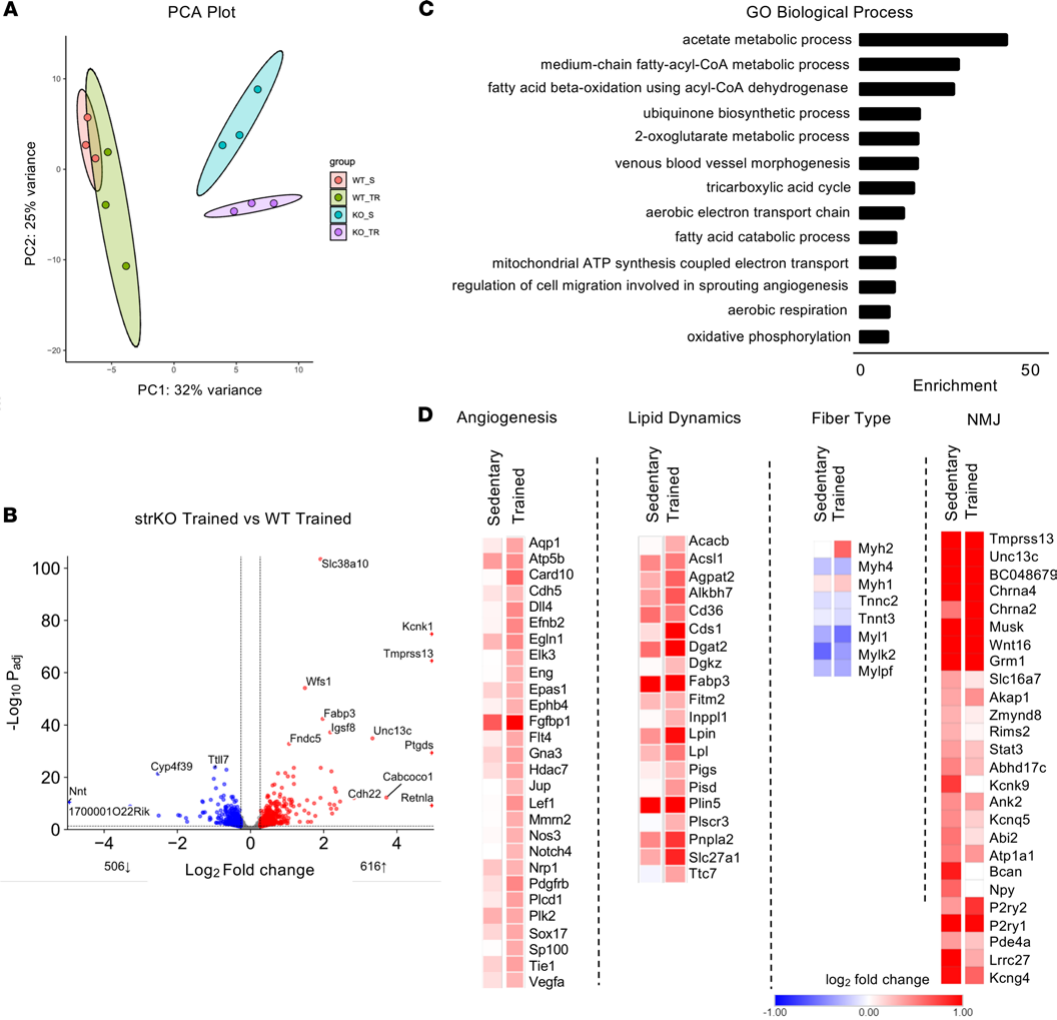
RIP140 regulates an array of pathways/processes known to be involved in muscle endurance. (**A**) PCA plot displaying WT control sedentary (WT_S), WT control trained (WT_TR), str*Nrip1*^–/–^ (strKO) sedentary (KO_S), and str*Nrip1*^–/–^ trained (KO_TR) groups from RNA-seq of EDL muscle (*n* = 3 per group). (**B**) Volcano plot comparing strKO trained and WT control trained differentially expressed genes (DEGs) from RNA-seq of EDL muscle. (**C**) Gene Ontology (GO) pathway analysis of upregulated Biological Process pathways in strKO trained EDL compared to WT control trained EDL. (**D**) Heatmap of upregulated DEGs at sedentary and trained conditions in strKO EDL muscle compared to corresponding WT controls. Fold change (FC) > 1.2, FDR < 0.05.

Previous studies have demonstrated that loss of RIP140 increases mitochondrial oxidative capacity in adipocytes (30, 31). In addition, we have shown that cardiac-specific deletion of RIP140 increases cardiomyocyte mitochondrial respiratory capacity and fatty acid oxidation rates (25). Concordantly, the robust endurance phenotype of the str*Nrip1^–/–^* and skm*Nrip1^–/–^* mice suggested that processes in addition to fuel and energy metabolism were induced in muscle of these mice. Indeed, a variety of cellular and structural processes are known to be transcriptionally regulated in response to endurance training such as fiber type, mitochondrial biogenesis, and angiogenesis (32). Given that RIP140 often serves as a transcriptional corepressor, we first sought to delineate the genes and pathways that were induced in str*Nrip1^–/–^* muscle. RNA-seq was conducted on EDL of all 4 groups in the training study: WT control sedentary, str*Nrip1^–/–^* sedentary, WT control trained, and str*Nrip1^–/–^* trained. Principal component analysis (PCA) displayed distinct separation between WT control and str*Nrip1^–/–^* groups (Figure 4A). The groups aligned largely along genotypes; WT control sedentary and WT control trained groups were distinct from str*Nrip1^–/–^* sedentary and str*Nrip1^–/–^* trained groups (Figure 4A). The impact of RIP140 on gene expression in the sedentary groups was first assessed. Differential expression analysis comparing str*Nrip1^–/–^* and WT control sedentary groups revealed 421 upregulated genes and 288 downregulated genes (fold change [FC] > 1.2 and FDR < 0.05) (Supplemental Figure 8A). As expected, Gene Ontology (GO) pathway analysis demonstrated upregulation of many energy metabolic processes, including mitochondrial respiration and electron transport, fatty acid β-oxidation, tricarboxylic acid cycle, and triglyceride/lipid droplet dynamics (Supplemental Figure 8B). Downregulated pathways included fast-twitch skeletal muscle fiber contraction, glucose catabolic process to lactate via pyruvate (glycolysis), and other glucose metabolic processes (Supplemental Figure 8C). Similar RNA-seq results were found in sedentary skm*Nrip1^–/–^* TA (Supplemental Figure 9, A–D). We next compared the gene expression profiles of str*Nrip1^–/–^* trained and WT control trained mice. This comparison identified 616 upregulated and 506 downregulated differentially expressed genes (DEGs) in str*Nrip1^–/–^* trained EDL compared with WT control trained EDL (FC > 1.2 and FDR < 0.05) (Figure 4B). GO pathway analysis of str*Nrip1^–/–^* trained EDL compared with WT control trained EDL revealed differential upregulation of many of the same genes and pathways that are upregulated in sedentary str*Nrip1^–/–^* EDL (Figure 4, C and D). Interestingly, several canonical non-metabolic muscle fitness genes were also upregulated in the sedentary and trained str*Nrip1^–/–^* muscle, including angiogenesis and NMJ biosynthesis (Figure 4D). These results provide evidence that RIP140 serves to repress genes involved in a larger network of muscle processes involved in the fitness phenotype, ranging from energy metabolism and structural adaptive responses, such as angiogenesis and NMJ remodeling.

**Figure 5:**
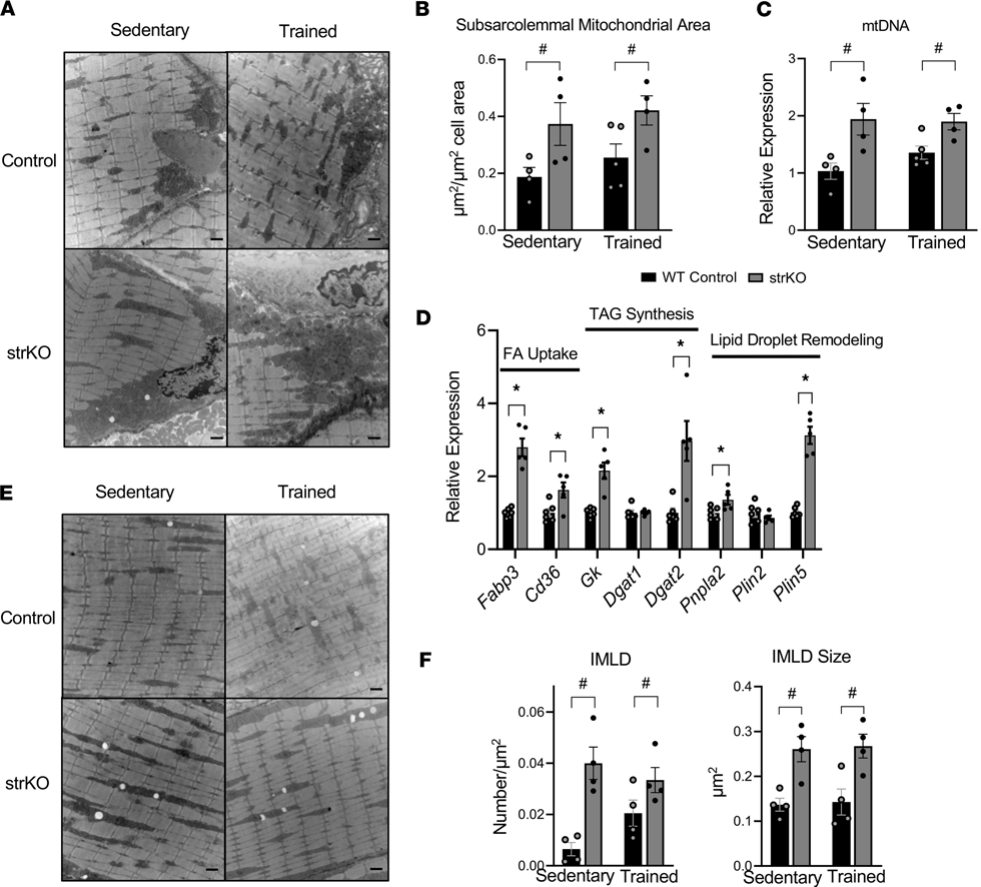
The ultrastructure of str*Nrip1*^–/–^ EDL reveals increased subsarcolemmal mitochondria and intramyocellular lipid droplets (IMLDs) in close apposition. (**A**) Representative electron micrographs of subsarcolemmal mitochondria in EDL muscle from 16-week-old male str*Nrip1*^–/–^ (strKO) sedentary, WT control sedentary, str*Nrip1*^–/–^ trained, and WT control trained mice. Scale bars: 1 μm. (**B**) Subsarcolemmal mitochondrial density quantified from the electron micrograph images from strKO sedentary, WT control sedentary, strKO trained, and WT control trained mice (*n* = 4–5 mice per group, 5 images analyzed per mouse). (**C**) Mitochondrial DNA (mtDNA) quantified from EDL muscle from male strKO sedentary, WT control sedentary, strKO trained, and WT control trained mice (*n* = 4–5 mice per group). (**D**) Expression of genes involved in triglyceride dynamics comparing strKO and WT control EDL muscle measured by qPCR (*n* = 5–6 mice per group). TAG, triacylglycerol; FA, fatty acid. (**E**) Representative electron micrographs of EDL muscle from male strKO sedentary, control sedentary, strKO trained, and WT control trained mice depicting lipid droplets (*n* = 4 mice per group). Scale bars: 1 μm. (**F**) IMLD number and size measured from the electron micrographs in EDL muscle from male strKO sedentary, control sedentary, strKO trained, and WT control trained mice (*n* = 4 per group, 5 images analyzed per mouse). Values are the mean ± SEM. ^#^*P* < 0.05 comparing genotypes and ^†^*P* < 0.05 comparing training by 2-way ANOVA displaying main effects (**B**, **C**, and **F**) or by 2-tailed, unpaired Student’s *t* test (**D**).

We sought to assess structural correlates of the activated gene regulatory pathways in str*Nrip1^–/–^* mice at baseline and with training, starting with the well-known adaptive increase in capacity for mitochondrial energy transduction. Many of the genes upregulated in the muscle of the str*Nrip1^–/–^* mouse model are involved in mitochondrial fuel and energy transduction. Endurance training has been shown to induce mitochondrial biogenesis in rodent and human skeletal muscle (33–36). Quantitative electron microscopy analyses demonstrated significantly increased subsarcolemmal mitochondrial area in str*Nrip1^–/–^* EDL at baseline compared with WT controls, which was not significantly increased with training (Figure 5, A and B). This corresponded to EDL mitochondrial DNA (mtDNA) content (Figure 5C). Measurement of intermyofibrillar (IMF) mitochondria displayed an increase in str*Nrip1^–/–^* trained EDL compared with sedentary str*Nrip1^–/–^* EDL (str*Nrip1^–/–^* trained: 0.060 ± 0.02; str*Nrip1^–/–^* sedentary: 0.022 ± 0.004; *P* = 0.03), while all other group comparisons showed no significant difference (WT trained: 0.046 ± 0.014; WT sedentary: 0.039 ± 0.01).

Several highly regulated transcriptional pathways in the str*Nrip1^–/–^* EDL involve triglyceride and lipid droplet dynamics, including fatty acid uptake, lipid biosynthetic processes, and lipid droplet remodeling (Figure 5D). This is interesting given that with endurance exercise training, an increase in lipid droplet storage occurs, presumably to meet the increased demand for mitochondrial fatty acid oxidation (37, 38). Electron microscopy revealed occasional intramyocellular lipid droplets (IMLDs) in contact with IMF mitochondria in sedentary WT control EDL (Figure 5E). In contrast, a significantly greater number of lipid droplets, juxtaposed to IMF mitochondria, were observed in sedentary str*Nrip1^–/–^* muscle (Figure 5E). Quantification of the lipid droplets in contact with IMF mitochondria confirmed a significantly greater number of lipid droplets in str*Nrip1^–/–^* muscle compared with WT muscle (Figure 5F). Average lipid droplet size was also larger in str*Nrip1^–/–^* muscle compared with WT control muscle (Figure 5F). Training did not significantly alter lipid droplet number or size (Figure 5F). These collective results provide structural correlates for the induction of genes involved in fatty acid storage and lipolysis as well as an increase in mitochondrial capacity for oxidation in str*Nrip1^–/–^* muscle, consistent with the observed endurance phenotype.

**Figure 6:**
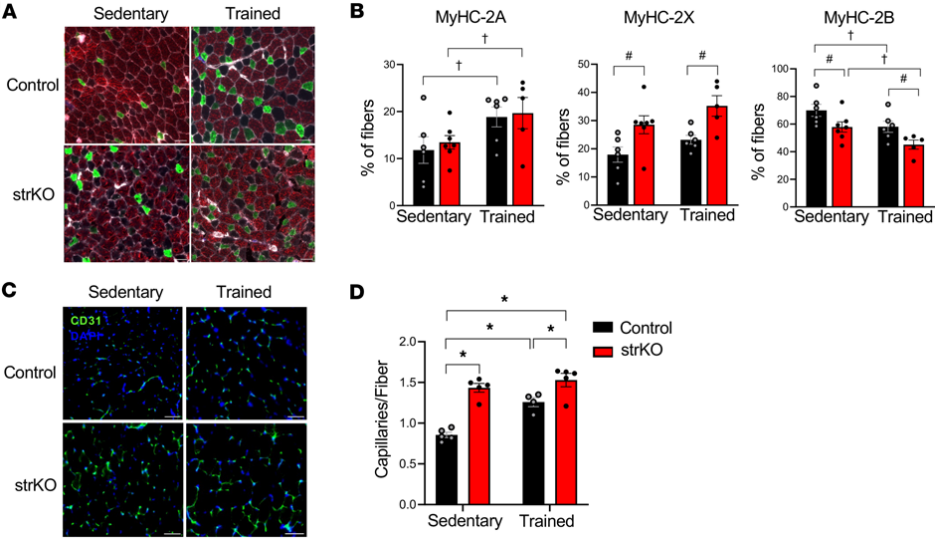
str*Nrip1*^–/–^ mice display a shift in fiber type and increased angiogenesis. (**A**) Fiber type staining of EDL muscle from 16-week-old male str*Nrip1*^–/–^ (strKO) sedentary, control sedentary, str*Nrip1*^–/–^ trained, and control trained mice (*n* = 5–7 mice per group). Scale bars: 50 μm. (**B**) Quantification of myosin expression (MyHC-2A, MyHC-2X, and MyHC-2B) from EDL fiber type images of male strKO sedentary, control sedentary, strKO trained, and control trained mice (*n* = 5–7 mice per group). (**C**) CD31 staining (green) of EDL muscle from 16-week-old male strKO sedentary, control sedentary, strKO trained, and control trained mice (*n* = 4–5 mice per group). Scale bars: 50 μm. (**D**) Quantification of capillaries per fiber from CD31-immunostained EDL images from strKO sedentary, control sedentary, strKO trained, and control trained mice (*n* = 4–5 mice per group). Values are the mean ± SEM. ^#^*P* < 0.05 comparing genotypes and ^†^*P* < 0.05 comparing training by 2-way ANOVA displaying main effects (**B**) or *P < 0.05 by 2-way ANOVA with Tukey’s multiple-comparison test (**D**).

Canonical changes in fiber type involving a shift from fast glycolytic fibers (MyHC-2B) to intermediate and oxidative fibers (MyHC-2X, MyHC-2A, and MyHC-1) occur in endurance-trained muscle (39, 40). Consistent with such a shift, the gene expression profiling indicated that expression of a subset of fast intermediate gene isoforms was increased and fast glycolytic isoforms decreased in sedentary str*Nrip1^–/–^* muscle (Figure 4D). Fiber type composition was determined using myosin immunostaining in str*Nrip1^–/–^* muscle. A significant increase in type 2X fibers (MyHC-2X) and a decrease in type 2B fibers (MyHC-2B) was observed in str*Nrip1^–/–^* EDL at baseline compared with WT controls (Figure 6, A and B). Training induced an increase in type 2A fibers (MyHC-2A) in both genotypes along with a further decrease in type 2B fibers (Figure 6, A and B). The fiber type shift patterns were similar in a second fast glycolytic muscle, TA muscle, with a trend toward increased type 2A fibers at baseline in str*Nrip1^–/–^* mice compared with WT control sedentary mice (Supplemental Figure 10, A and B). Taken together, these results indicate that fast-twitch muscles of the str*Nrip1^–/–^* mice display a shift away from the fast glycolytic fiber type (type 2B) toward intermediate (type 2X) and oxidative (type 2A) fibers.

Skeletal muscle capillary density increases in response to endurance training as an adaptation for higher oxygen delivery for aerobic respiration (41–43). Notably, expression of a subset of angiogenic genes (*Vegfa*, *Atp5b*, *Egln1*, *Epas1*, *Fgfbp1*, *Tie1*, and *Plk2*) was increased in str*Nrip1^–/–^* EDL at baseline, with an even greater number increased with training (Figure 4D). Capillary density was assessed via CD31 immunostaining, a marker of endothelial cells, in muscles from all 4 groups of the training study. CD31 staining of EDL and TA muscle revealed an increase in capillary number per muscle fiber in sedentary str*Nrip1^–/–^* mice compared with WT control mice (Figure 6, C and D, and Supplemental Figure 10, C and D). Training further increased capillary number in the str*Nrip1^–/–^* TA and EDL (Figure 6, C and D, and Supplemental Figure 10, C and D). These results indicate str*Nrip1^–/–^* muscle displays increased angiogenesis compared with WT controls and is potentiated with training.

**Figure 7:**
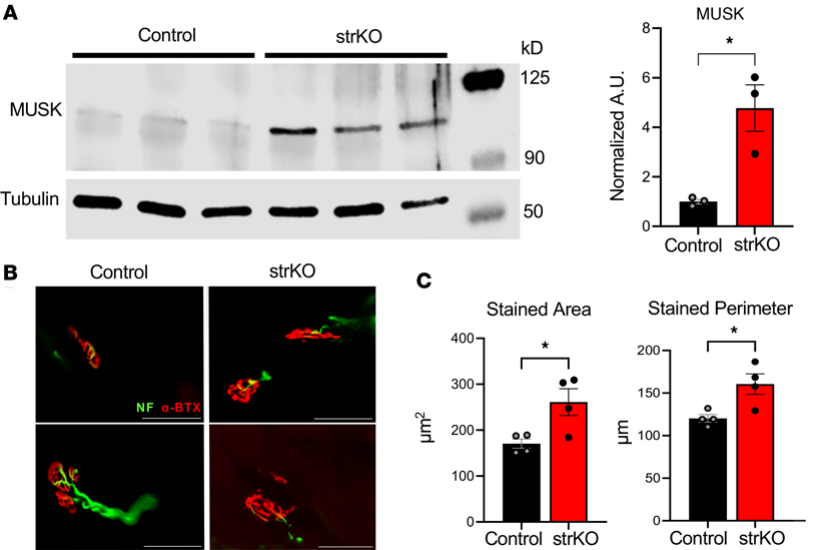
Neuromuscular junction (NMJ) remodeling in sedentary str*Nrip1*^–/–^ mouse muscle. (**A**) MUSK Western blot comparing WT control and str*Nrip1*^–/–^ (strKO) EDL muscle and quantification (normalized to tubulin) from 8-week-old male mice (*n* = 3 mice per group). (**B**) α-Bungarotoxin (α-BTX; motor end plate, red) and neurofilament (NF; nerve terminal, green) staining in WT control and strKO EDL muscle from 8-week-old male mice. Scale bars: 50 μm. (**C**) Stained area and stained perimeter measured from α-BTX (motor end plate) staining from WT control and strKO EDL muscle (*n* = 4 mice per group, 12–15 NMJs measured per mouse). Values are the mean ± SEM. *P < 0.05 versus control by 2-tailed, unpaired Student’s *t* test (**A** and **C**).

The NMJ is known to undergo adaptive remodeling in response to endurance training, including an increase in the area and density of clustered acetylcholine receptors within the motor end plate (44–48). A group of genes involved in NMJ biogenesis were significantly upregulated in sedentary and trained str*Nrip1^–/–^* EDL, including *Musk*, *Chrna4*, *Chrna2*, *Grm1*, *Zmynd8*, *Unc13c*, *Wnt16*, and *Pde4a* (Figure 4D) (49–51). One of the most highly regulated genes was muscle-specific kinase, or *Musk*, a tyrosine kinase responsible for clustering of the acetylcholine receptors at the motor end plate (52–54). Immunoblotting confirmed increased MUSK protein levels in str*Nrip1^–/–^* EDL muscle (Figure 7A). Given the role MUSK plays in acetylcholine receptor clustering at the motor end plate, we next examined the acetylcholine receptor density in str*Nrip1^–/–^* and WT control EDL muscle using α-bungarotoxin (α-BTX) immunostaining. The area and perimeter of α-BTX staining was significantly increased in sedentary str*Nrip1^–/–^* EDL muscle compared with WT control EDL muscle (Figure 7, B and C). These results demonstrate that sedentary str*Nrip1^–/–^* mice exhibit NMJ remodeling in fast-twitch muscle, akin to what occurs with exercise training.

### RIP140 regulates the transcription of a network of muscle genes involved in NMJ biosynthesis and remodeling.

**Figure 8:**
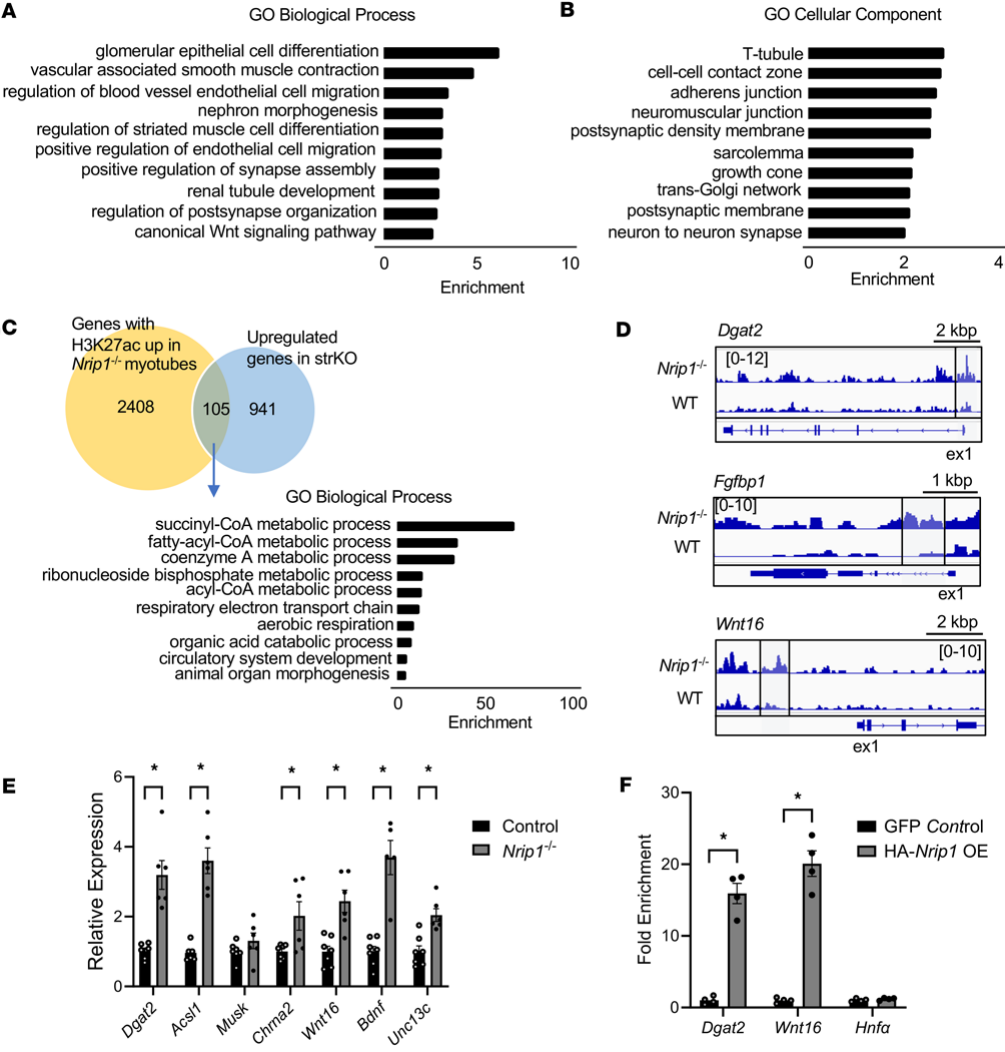
CUT&RUN sequencing reveals RIP140 targets involved in mitochondrial function, angiogenesis, and the NMJ. (**A**) GO Biological Process pathways and (**B**) Cellular Component pathways based on analysis of genes near increased H3K27ac peaks in *Nrip1*^–/–^ myotubes compared to WT control primary myotubes. (**C**) Intersection analysis of upregulated H3K27ac gene annotated peaks in *Nrip1*^–/–^ myotubes and upregulated differentially expressed genes (DEGs) in strKO gastrocnemius compared to their corresponding controls. GO biological process pathways are represented from the intersected gene list. (**D**) Representative CUT&RUN sequencing browser tracks from *Dgat2*, *Fgfbp1*, and *Wnt16* comparing *Nrip1*^–/–^ and WT control. Browser tracks were obtained utilizing the Integrative Genomics Viewer (https://igv.org/) displayed as sequence reads mapped to the reference genome (mouse mm10). The gene locus is represented in the bottom segment of each browser track and exon 1 (ex. 1) is labeled. Sequence read peaks are represented on a log scale. (**E**) Gene expression of fatty acid metabolic and NMJ genes denoted at the bottom comparing *Nrip1*^–/–^ and WT control myotubes (*n* = 6–7 samples per group). (**F**) ChIP-qPCR enrichment at *Dgat2* and *Wnt16* H3K27ac peaks identified by CUT&RUN analyses and *Hnfa* (negative control) in 3x-HA-Nrip1–overexpressing (OE) and GFP control C2C12 myotubes (*n* = 4 samples per group). Values are the mean ± SEM. *P < 0.05 versus control by 2-tailed, unpaired Student’s *t* test (**E** and **F**).

To further assess the direct transcriptional control of muscle RIP140 gene targets, we performed CUT&RUN sequencing using an antibody against the known histone enhancer mark, H3K27ac, in *Nrip1^–/–^* and WT control primary mouse myotubes isolated from gastrocnemius muscle. H3K27ac peaks that were significantly increased in *Nrip1^–/–^* myotubes compared with WT control myotubes were identified and the nearest genes were annotated. GO analysis of the annotated nearest genes demonstrated enrichment in biological process pathways involved in muscle, vascular, and neuronal development, including striated muscle cell differentiation, endothelial cell migration, and regulation of NMJ assembly (Figure 8, A and B). Intersection of this dataset with DEGs identified within an in vivo RNA-seq dataset of *Nrip1^–/–^* and WT control gastrocnemius muscle (25) identified overlap of 105 genes (Figure 8C). GO biological process pathway analysis of the intersected genes revealed pathway enrichment in metabolic (fatty-acyl-CoA metabolic process and respiratory electron transport chain), and developmental (circulatory system development and animal organ morphogenesis) processes (Figure 8C). Metabolic genes included a subset involved in fatty acid metabolism, including *Acsl1*, *Dgat2*, and *Echs1*, known targets of PPARα, a nuclear receptor known to be modulated by RIP140 (23, 55–57). Developmental pathway genes included those involved in angiogenesis (*Fgfbp1*, *Esm1*, and *Epas1*) and NMJ formation (*Wnt16*, *Stat3*, *Bdnf*, *Unc13c*, *Slc16a7*, and *Abhd17c*). Representative genomic browser shots displaying the CUT&RUN sequences mapped to the mouse genome comparing *Nrip1^–/–^* and control groups showing the differences in H3K27ac deposition for representative genes are shown in Figure 8D.

The identification of the genes involved in NMJ biogenesis was of particular interest given the observation that the RIP140-deficient muscle exhibited an expansion of NMJ structure and less is known about the gene regulation of this process compared with the metabolic pathways. Increased expression of *Wnt16*, *Chrna2*, *Bdnf*, and *Unc13c* was confirmed in *Nrip1^–/–^* myotubes along with the expected upregulation of the lipid metabolic genes *Dgat2* and *Acsl1* (Figure 8E). In contrast with the other NMJ genes, the expression of *Musk* was not increased in *Nrip1^–/–^* myotubes, concordant with no change in H3K27ac deposition (Figure 8E). This was surprising given the significant induction of *Musk* mRNA and protein levels in *Nrip1^–/–^* EDL muscle. We next sought to determine whether the regulation of the NMJ program was a direct effect of transcriptional regulation by RIP140. We focused on Wnt16 because it has been implicated in the process of acetylcholine receptor clustering (49). Given that antibodies available for RIP140 are not suitable for ChIP, ChIP-qPCR was performed on 3x-HA-*Nrip1*–overexpressing C2C12 myotubes compared to backbone vector–infected control myotubes utilizing an anti-HA antibody. As expected, RIP140 occupation was confirmed on the *Dgat2* promoter within the H3K27ac peak region (Figure 8F). Notably, this region contains a known PPAR binding motif (58). Importantly, direct occupation of RIP140 within the *Wnt16* H3K27ac deposition region was also demonstrated (Figure 8F). These results indicate that Wnt16 is a direct target of RIP140 and likely plays a key role upstream of NMJ biogenesis.

## Discussion

Transcriptional regulatory mechanisms driving muscle endurance fitness have begun to be unraveled over the past decade. The exercise-inducible transcriptional coregulator, PGC-1α, and its downstream nuclear receptors, ERR and PPAR, were shown to be necessary for the increase in skeletal muscle mitochondrial fatty acid oxidation and respiration capacity with endurance exercise training (59–61). Here, we demonstrate the impact of inhibiting a natural “brake” on this exercise-induced transcriptional regulatory axis, the coregulator RIP140, in mouse skeletal muscle. We found that skeletal muscle–specific depletion of RIP140 in mice triggers an array of downstream metabolic and structural responses that comprise an enhanced endurance phenotype in the absence of exercise training. In addition to the augmentation of muscle oxidative metabolism, as might be predicted based on the function of RIP140 in other tissues, downstream structural changes were observed in RIP140-deficient muscle, including angiogenesis and expansion of the NMJ. These findings were most pronounced in fast-twitch, glycolytic muscles where *Nrip1* expression is higher compared with oxidative muscles.

An increase in the capacity for mitochondrial respiration and oxidation of fatty acids to generate ATP is a well-defined adaptive response of skeletal muscle to endurance exercise training. The RIP140-deficient state resulted in an augmentation of muscle oxidative capacity, including mitochondrial biogenesis, induction of fatty acid oxidation genes, and expansion of IMLDs. The high capacity to oxidize fat in *Nrip1^–/–^* mice was demonstrated by the maintenance of a low RER and high percentage of fatty acid oxidation throughout the exercise period. However, muscle adaptations beyond fuel utilization are required for enhanced endurance performance. Indeed, structural changes occurred in the RIP140-deficient muscle, including a shift to more oxidative muscle fiber types and an angiogenic response. These changes may be mediated by the nuclear receptors PPAR and ERR, given RIP140 has been shown to modulate these transcription factors in other cell types. In addition, similar effects have been shown with transgenic overexpression of PPAR and ERR (10, 11).

An interesting finding in this study was the observation of expansive NMJ remodeling in the RIP140-deficient muscle. Expansion of the NMJ occurs with exercise training, including increased density and clustering of acetylcholine receptors at the motor end plate (44, 45, 47, 62). Conversely, the density of the acetylcholine receptors at the motor end plate decreases and fragments with aging and muscular dystrophies (63, 64). We found that NMJ genes involved in formation and maintenance of the motor end plate were upregulated in RIP140-deficient muscle, including *Musk*, *Chrna4*, *Chrna2*, and *Wnt16*. Staining of acetylcholine receptors in str*Nrip1^–/–^* EDL muscle demonstrated increased density and area of the motor end plate. Our integrated CUT&RUN and RNA-seq results identified genes near upregulated H3K27ac peaks in *Nrip^–/–^* muscle that are known to be involved in the formation of the motor end plate. This analysis identified *Wnt16* as a gene of interest. *Wnt16* is downstream of a region of increased H3K27ac deposition and is expressed at increased levels in *Nrip1^–/–^* primary myotubes and *Nrip1^–/–^* skeletal muscle in vivo. Members of the Wnt family, including Wnt16b, have been implicated in NMJ biosynthesis and acetylcholine receptor clustering at the motor end plate (49). Taken together, our results indicate that RIP140 serves as a repressor of *Wnt16* transcription. However, the transcription factor targets involved in this regulation remain unknown. Notably, overexpression of PGC-1α in muscle has been shown to upregulate a subset of NMJ genes in mice, a mechanism that is dependent on the transcription factor GABPA (50). Several putative GABPA binding sites are present within the genomic region that RIP40 occupies on *Wnt16*, suggesting that RIP140 may modulate the PGC-1/GABP axis (65). Further delineation of the mechanism whereby RIP140 deficiency activates *Wnt16* and downstream NMJ biogenesis will be of interest.

Our results raise the question of the normal physiological role of RIP140 in muscle and other tissues. Why is a repressor of muscle endurance machinery necessary? The answer to this question is unknown but several functions are possible. First, RIP140 can serve as a transcriptional coactivator or corepressor depending on cell context and developmental stage (19, 20, 66–70). It is possible that the most important function is during development where it has been shown to be involved in cellular proliferation and differentiation (71–76). Accordingly, perhaps the postnatal phenotype of RIP140-deficient muscle reflects changes during development. The strong enrichment of developmental programs in our CUT&RUN data are consistent with this hypothesis. A second possibility is that RIP140 serves a key physiologic homeostatic function in striated muscle by acting as a rheostat for endurance muscle function balanced against the need for recruitment of fast-twitch muscle for rapid sprinting. Depending on the environmental circumstance, demands for endurance performance versus “fight-or-flight” responses may be determined in part via coregulators such as RIP140. Consistent with this proposed role, *Nrip1* begins to appear in the evolutionary tree in organisms capable of extensive locomotion and travel across environments. *Nrip1* orthologs are present in *Xenopus* (gene ID: 100037834), zebrafish (gene ID: 796407), and mammals but not in lower organisms such as yeast, *Drosophila*, or *C*. *elegans* (sources mined by GeneCards [https://www.genecards.org/], updated Dec 25, 2024). Notably, the mechanisms involved in the regulation of RIP140 activity under physiological conditions are poorly understood. We have found no regulation of transcript levels in response to exercise training (Supplemental Figure 7D), which is consistent across the literature (77–79). However, RIP140 transcript and protein levels are induced in response to acute exercise, likely regulating oxidative versus glycolytic metabolism during exercise (77–79). It appears that RIP140 is more likely controlled at a posttranslational level, as suggested by several potential phosphorylation and acetylation sites (80–83). Defining the regulation of posttranslational modifications in response to exercise training will further elucidate the role of RIP140 in controlling muscle endurance.

Our results have translational implications. Physical inactivity related to chronic disease or primary muscle diseases leads to a vicious cycle of reduced muscle function and mass. The effects of targeting RIP140 in muscle suggest that inhibition of its corepressor function may prove to be a fruitful therapeutic avenue, particularly in conjunction with anabolic agents. In addition, the impact of RIP140 inhibition on converting glycolytic fibers to a more oxidative phenotype suggests that targeting this coregulator may be useful for chronic diseases disproportionately affecting fast-twitch muscle including Duchenne’s muscular dystrophy, age-related sarcopenia, and amyotrophic lateral sclerosis (84). The effects of RIP140 deficiency on neuromuscular remodeling could also translate to conditions that impact the motor end plate, including aging-induced fragmentation of the NMJ, recovery from muscle injury, and primary neuromyopathies.

Several caveats regarding translational potential should be noted. First, RIP140 is active in many tissues and has been shown to function both as a corepressor and coactivator, the latter often in developmental functions in females such as mammary gland development and ovulation through interaction with the estrogen receptor (66, 85, 86). Secondly, the phenotype described herein is based on prenatal disruption of the *Nrip1* gene. To date, we have had difficulty achieving an inducible postnatal *Nrip1* knockout using traditional approaches, which would be more relevant for studies aimed at the impact of RIP140 inhibition in disease states. Lastly, given that aging results in a reduction in strength related to reduced type 2 fibers, it is possible that RIP140 could further exacerbate this transition given its effects shift fast fibers to more oxidative fibers.

## Methods

### Sex as a biological variable.

Both male and female str*Nrip1^–/–^* and control mice were included in phenotype testing. Only male mice were utilized in further training studies due to a greater observed phenotype compared with females.

### General protocol for mouse studies.

Mouse studies were performed on male or female mice 8–16 weeks of age. Mice were housed in a facility with a 12-hour light/12-hour dark cycle. Tissues were harvested between 12 and 3 pm. Mice were anesthetized with pentobarbital (Sagent; 100 mg/kg) prior to tissue removal. In some cases, separate cohorts of mice were utilized for physiological testing and other endpoints due to the need for sacrifice for the measurement or for practical reasons related to fatigue or stress that can confound the interpretation of the second assessment.

### Generation of mouse models.

*Nrip1^fl/fl^* mice (C57BL/6J) were generated and provided by Zhenji Gan (National Resource Center for Mutant Mice, Model Animal Research Center of Nanjing University, Nanjing, China) (25). Briefly, *Nrip1^fl/fl^* mice were generated via CRISPR/Cas9 targeting. The *Nrip1* (RIP140) gene has 4 exons, with the ATG start codon in exon 4 and TAA stop codon in exon 4. Cas9 mRNA, sgRNA, and donor were coinjected into zygotes. sgRNA directed Cas9 endonuclease cleavage targeted introns 3–4, downstream of the 3′-UTR, to create a double-strand break. This double-strand break was repaired and resulted in *loxP* sites inserted into introns 3–4 and downstream of the 3′-UTR by homologous recombination. The *Nrip1^fl/fl^* mice were bred with C57BL/6JN mice expressing Cre recombinase under control of either the MCK (The Jackson Laboratory, stock 006475) or myogenin (29) promoter to generate str*Nrip1^–/–^* and skm*Nrip1^–/–^* RIP140-deficient mice. Male and female littermate mice were genotyped as Cre-positive or Cre-negative (littermate controls) and group housed.

### Endurance performance treadmill testing.

Mice were acclimated for 2 days at 10 m/min for 9 minutes with a 5° incline and then the speed was increased to 20 m/min for 1 minute for a total of 10 minutes. Mice that did not run by the second acclimation day were deemed non-runners and removed from the study. On day 3, the mice were subjected to running for 10 minutes at 10 m/min at 5° incline. At 10 minutes, the speed increased by 2 m/min for 15 minutes, and then increased 2 m/min every 15 minutes until exhaustion. Exhaustion was deemed as 5 consecutive attempts to keep the mouse on the treadmill without any effort given by the mouse or spending over 50% of running time at the back of the treadmill (10).

### VO_2max_ testing.

Mice were exercised in an enclosed treadmill connected by tubes to a CLAMS (Colombus Instruments) machine. Mice were acclimatized to the motorized treadmill prior to the procedure. For the VO_2max_ protocol, mice were exposed to the treadmill for 5 minutes at 0 m/min. The VO_2max_ protocol was as follows: 5 m/min at a 5° incline for 2 minutes, 20 m/min at 10° incline for 2 minutes, 22 m/min at 10° incline for 2 minutes, 24 m/min at a 15° incline for 2 minutes, 26 m/min at 20° incline for 2 minutes, 28 m/min at 25° incline for 2 minutes, and increasing 2 m/min at this incline every 2 minutes until exhaustion. Exhaustion was defined as greater than 20 seconds on the shock grid without treadmill reengagement or spending greater than 50% of their time on the shock grid. VCO_2_ and VO_2_ readings were taken every 15 seconds of the protocol in which VO_2max_ and RER values could be obtained, as previously described (36, 87). Fatty acid and carbohydrate oxidation percentages were calculated as previously described (88). Anaerobic threshold was demonstrated by the crossover of fatty acid oxidation to a sharp increase in carbohydrate oxidation (88).

### RNA isolation and qRT-PCR.

Total RNA was isolated using the RNeasy Mini Kit (Qiagen) following the manufacturer’s protocol. dsDNA was made from extracted RNA using the Affinity Script cDNA synthesis kit (Agilent Technologies) using 0.5 μg RNA. Brilliant III Ultra-Fast SYBR Green QPCR Master Mix (Agilent Technologies) was used to perform qPCR reactions on a QuantStudio 6 Flex Real-Time PCR System (Applied Biosystems) using specific primer sets designed for each gene. Primer sets are listed in Supplemental Table 1. All target mRNA expression was normalized to *Rplp0* (36B4).

### Ex vivo muscle physiological analysis.

The Aurora Mouse 1200A System equipped with Dynamic Muscle Control v.5.415 software was utilized for all measurements, as previously described (89). EDL and soleus muscles were maintained in oxygenated Ringer’s solution (100 mM NaCl, 4.7 mM KCl, 3.4 mM CaCl_2_, 1.2 mM KH_2_PO_4_, 1.2 mM MgSO_4_, 25 mM HEPES, and 5.5 mM D-glucose) at 24°C. For maximal tetanic measurements, a stimulus was repeated at 120 Hz (EDL) or 80 Hz (soleus) for 500 ms. Fatigue measurements were performed 5 minutes after the last tetanic stimulation. Fatigue was induced by stimulation every second for 5 minutes using 40-Hz pulses lasting 330 ms. Recovery was determined after a 5-minute rest following the fatigue protocol. Six tetanic measurements were performed with 2 minutes of rest between each stimulus. Recovery was determined by the tetanic force generated after the fatigue protocol divided by the initial tetanic force represented as a percentage. The muscle cross-sectional area (CSA) was determined by dividing muscle mass by the product of the muscle density coefficient (1.06 g/cm^3^), optimal muscle fiber length for producing muscle optimal length (*L*_0_), and the fiber length coefficient (0.45 for EDL, 0.69 for soleus). Maximum isometric tetanic force measurements were normalized to CSA to determine specific force.

### Mouse voluntary wheel training.

Mice at 8 weeks of age were subject to voluntary wheel running for 8 weeks using wire wheels and pedometer trackers (36). Mice that ran less than 5 km/night after the first adjustment week were removed from the study and deemed non-runners. Wheels were removed from the cages for 48 hours prior to any exercise testing or other testing endpoints.

### Electron microscopy preparation.

Tissues for electron microscopic examination were fixed with 2.5% glutaraldehyde and 2.0% paraformaldehyde in 0.1 M sodium cacodylate buffer, pH 7.4, overnight at 4°C. After subsequent buffer washes, the samples were postfixed in 2.0% osmium tetroxide with 1.5% K_3_Fe(CN)_6_ for 1 hour at room temperature and rinsed in DH_2_O. After dehydration through a graded ethanol series, the tissues were infiltrated and embedded in EMbed-812 (Electron Microscopy Sciences). Sections were stained with uranyl acetate and SATO lead and examined with a JEOL 1010 electron microscope fitted with a Hamamatsu digital camera and AMT Advantage Nano Sprint 500 software.

### Quantification of mitochondria and lipid droplets.

The density of mitochondria within the myocytes was expressed as μm^2^ area occupied by mitochondria/μm^2^ myocyte and as number of mitochondria per μm^2^ myocyte (90, 91). Images at relatively high and low magnifications were used from areas rich in subsarcolemmal or IMF mitochondria. The mitochondria and cell areas were assessed using ImageJ (NIH) (92). The perimeter of the cell and mitochondria areas were traced and computed. Lipid droplets were measured in a similar manner and represented by number per cell area and lipid size.

### Histology.

TA, EDL, and soleus muscle were harvested and immersed in gum tragacanth and frozen on a histology block in liquid nitrogen–cooled isopentane. Muscle cross or longitudinal sections were cut on a Leica cryostat at either 10, 14, or 20 μm. Slides were imaged using a Zeiss widefield microscope.

SDH staining was performed on 14-μm frozen muscle sections. Nitro blue tetrazolium (NBT) was prepared in 0.2 M phosphate buffer and incubated on the muscle sections for 30 minutes at 37°C. Unbound NBT was removed by exchanges of 30%, 60%, and 90% acetone in ascending order. Slides were mounted with aqueous mounting medium.

Fiber type staining was performed using 10-μm frozen sections, as previously described (93). The following myosin heavy chain antibodies were purchased from the Development Studies Hybridoma Bank (DSHB): MyHC-1 (BA-D5), MyHC-2A (SC-71), MyHC-2X (unstained), and MyHC-2B (BF-F3). Secondary antibodies were purchased from Jackson ImmunoResearch: goat anti-mouse IgG2b DyLight 405 (for BA-D5; 115-475-207); goat anti-mouse IgG1 Alexa Fluor 488 (for SC-71; 115-545-205); and goat anti-mouse IgM Alexa Fluor 594 (for BF-F3; 115-585-020). Fiber type analysis was performed by ImageJ utilizing the Myosight program to identify the predominant myosin expressed in each fiber (94).

CD31 staining was performed using 10-μm frozen sections fixed with 10% neutral-buffered formalin. CD31 antibody (CM303A, Biocare) was used at a 1:50 dilution. VECTASHIELD Vibrance Antifade Mounting Medium with DAPI (H-1800, Vector Laboratories) was used for DAPI staining. Capillaries per fiber were measured in ImageJ.

α-BTX staining was performed on 20-μm longitudinal sections. Sections were fixed in 4% paraformaldehyde and permeabilized with 0.1% Triton X-100/5% BSA. Sections were incubated with anti-neurofilament (2H3, DSBH) and anti–α-BTX (B13423, Thermo Fisher Scientific) at 1:1000 dilutions. Slides were analyzed using ImageJ as previously described (46).

### RNA-seq library preparation and sequencing.

The EDL muscle was isolated from an independent cohort of mice from the training study that was not subject to additional testing. RNA library preparations and sequencing were conducted at AZENTA. RNA integrity was measured prior to submission using Agilent TapeStation 4200 (Agilent Technologies). RNA-seq libraries were created utilizing the NEBNext Ultra RNA Library Prep Kit for Illumina (New England BioLabs). The sequencing library was quantified using a Qubit 2.0 Fluorometer (Life Technologies) and validated on the Agilent TapeStation (Agilent Technologies). The sequencing libraries were clustered on a single lane of a flow cell and loaded onto the Illumina HiSeq instrument (model 4000 or equivalent) according to manufacturer’s instructions. The samples were sequenced using a 2 × 150-bp paired-end configuration. HiSeq Control Software was utilized for image analysis and base calling. Raw sequence data generated by the Illumina HiSeq were converted into fastq files and demultiplexed using Illumina’s bcl2fastq 2.17 software.

### RNA-seq data processing and differential expression analysis.

Raw RNA-seq reads were quantified using Salmon (v1.10.2) (95) with the mm10 reference genome. The transcript-level quantifications were summarized to gene-level counts using tximport in R.

Differential gene expression analysis was performed using DESeq2 (v1.44.0) (96). Genes with an adjusted *P* value (FDR) of 0.05 or less and an absolute fold change of at least 1.2 were considered DEGs.

### Quantification of mtDNA.

Genomic/mtDNA was isolated using QIAzol (Qiagen). Back extraction was performed using 4 mol/L guanidine thiocyanate, 50 mM sodium citrate, and 1 mol/L Tris followed by isopropanol DNA precipitation, as previously described (25). mtDNA content was analyzed by SYBR green (Stratagene) using primers to identify NADH dehydrogenase subunit 1 (*mt-Nd1*), encoded in mtDNA, and lipoprotein lipase (*Ldl*), encoded in genomic DNA. *mt-Nd1* expression levels were normalized to *Ldl* expression levels to determine mtDNA content.

### Immunoblot analysis.

SDS-PAGE was performed on whole tissue or nuclear protein lysates and transferred to a nitrocellulose membrane, as previously described (97). Whole tissue lysates were extracted using RIPA buffer (50 mM Tris-HCl pH 7.4, 150 mM NaCl, 1 mM EDTA, 1% Triton X-100, 0.1% NP-40). Nuclear lysates were extracted using NE-PER Nuclear and Cytoplasmic Extraction Reagents (Thermo Fisher Scientific). Primary antibodies used were as follows: MUSK (Invitrogen, PA1-1741; 1:1000 dilution); RIP140 (Santa Cruz Biotechnology, sc-81370; 1:200 dilution); α-tubulin (Cell Signaling Technology, 3873; 1:5000 dilution); and lamin A/C (Cell Signaling Technology, 4777; 1:1000 dilution). Western blots were quantified using ImageStudio software (LICORbio).

### Satellite cell isolation and culture.

Satellite cells were isolated from the gastrocnemius muscles. Briefly, the muscle was incubated in collagenase type II (Life Technologies) and Dispase type II (Sigma-Aldrich) until most muscle was digested. The digested solution was passed through a cell strainer. Cells were plated in preplating medium (1 g/L glucose DMEM, 10% FBS, 1× Pen/Strep) on non-coated plates for 3 hours to remove non-satellite cells. Satellite cells were plated in growth media (Ham’s F-10, 20% FBS, 10 ng/mL EGF, 1 μg/mL insulin, 0.39 μg/mL dexamethasone, 1× Pen/Strep) and expanded. Two additional preplating steps were performed to remove non-satellite cells. Differentiation was performed between passages 6 and 9. Cells were differentiated in differentiation media (1 g/L glucose DMEM, 2% horse serum, 1× Pen/Strep) for 4 days before experimentation.

### CUT&RUN sequencing.

CUT&RUN sequencing was completed using the CUTANA ChIC/CUT&RUN Kit Version 4 (EpiCypher, New England Biolabs) following the manufacturer’s instructions. Briefly, nuclei were isolated from primary myotubes following the nuclei extraction protocol. Nuclei were pooled and split between antibody conditions. Nuclei were bound to ConA beads and incubated overnight with antibody (H3K27ac, Cell Signaling Technology, 4353; IgG, 13-0042, EpiCypher). The next day, nuclei were permeabilized, treated with 2.5 μL pAG-MNase and incubated at 4°C for 1 hour. For chromatin digestion, 1 μL of 100 mM calcium chloride was added and incubated at 4°C for 2 hours. DNA was purified and library preparation was performed following manufacturer’s instructions using the CUTANA CUT&RUN Library Prep Kit. Briefly, 5 ng of eluted DNA was end repaired and subjected to the protocol’s thermocycler program. Adapter ligation for Illumina followed by U-excision was completed and unique combinations of i5 and i7 indexing primers were used to amplify during PCR. PCR cleanup was completed, and samples were measured for concentration using a Qubit 2.0 Fluorometer. Samples were submitted to AZENTA and sequenced as described for RNA-seq.

### CUT&RUN data processing and peak analysis.

CUT&RUN sequencing data for H3K27Ac histone modifications were processed using a standardized pipeline. Paired-end reads were aligned to the mm10 reference genome using BWA-MEM2 (v2.2.1) (98) with default settings. Broad peak calling was performed using HOMER (v4.11) (99) to identify regions enriched for H3K27Ac modifications. Peaks were annotated with their nearest genes using HOMER’s annotation tools. To identify differentially acetylated regions between control and KO samples, HOMER was used with a 2-fold change or greater cutoff for peak intensity differences. Differential peaks were then mapped to their nearest genes, which were cross-referenced with the differential gene expression data from the RNA-seq analysis to assess transcriptional changes associated with H3K27Ac modifications.

### Generation of stable HA-Nrip1 C2C12 cell line.

HA-RIP140 lentivirus was generated by transfecting subconfluent C2C12 cells (ATCC) cultured in a gelatin-coated T25 flask with Lipofectamine 2000 (Thermo Fisher Scientific) according to the manufacturer’s instructions and the following plasmids: 3 μg psPAX2, 2 μg pMDG.2, and 4 μg of pLVX-3XHA-RIP140. The next day, medium was exchanged for DMEM containing 30% FBS. After 48 hours, medium was harvested and stored at 4°C, replacing with fresh DMEM supplemented with 30% FBS. The next day, the medium was harvested, pooled, and virus concentrated using LentiX (Takara) according to the manufacturer’s instructions. The viral pellet was resuspended in 300 μL PBS and stored at –80°C. Stable HA-RIP140 C2C12 cells were generated by adding 100 μL of concentrated lentivirus to subconfluent C2C12 cells cultured in a 6-well plate with DMEM, 10% FBS, and 8 μg/mL polybrene. The next day, cells were selected for stable plasmid integration with DMEM, 10% FBS, and 3 μg/mL puromycin. Selection media were exchanged daily until all non-infected control cells were dead.

### ChIP-qPCR.

ChIP was performed as previously described (100). Briefly, differentiated C2C12 myotubes were crosslinked with disuccinimidyl glutarate (DSG) for 45 minutes. DSG was removed and additional crosslinking performed using formaldehyde for 10 minutes. Cells were harvested and lysed. Lysates were sonicated using a Covaris sonicator. Proteins were immunoprecipitated using anti-HA tag (Proteintech, 51064-2-AP) overnight at 4°C. Purification of DNA fragments was completed using the QIAquick PCR purification kit (Qiagen). ChIP-qPCR primers are displayed in Supplemental Table 2.

### Statistics.

A Student’s *t* test was used for all 2-group comparisons when data were normally distributed based on a Shapiro-Wilk test (α = 0.05). We used 2-way ANOVA to analyze the effect of training and RIP140 deficiency on each dependent variable. When the interaction was found significant at a *P* value of less than 0.05, we explored simple effects with Tukey’s post hoc comparisons of the 4 group means. GraphPad Prism was used for graphical and statistical analysis (version 10.2.3).

### Study approval.

All animal studies were performed in accordance with NIH *Guide for the Care and Use of Laboratory Animals* (National Academies Press, 2011) and approved by the IACUC at the University of Pennsylvania.

### Data availability.

All genomics data generated in this study have been deposited in NCBI’s Gene Expression Omnibus (GEO). The skm*Nrip1^–/–^* RNA-seq data are available under accession GSE289576. The str*Nrip1^–/–^* with training RNA-seq data are available under accession GSE289569. The H3K27Ac CUT&RUN data for *Nrip1^–/–^* primary skeletal muscle myotubes are available under accession GSE289567. Values for all data points in graphs are present in the Supporting Data Values file.

## Author contributions

EP and DPK conceptualized the project. EP conducted experiments. DMM conducted the mitochondrial density calculations. SMS generated and validated the C2C12 RIP140 stable overexpression line. BPS performed the Musk Westerns and analysis. KB specifically conducted bioinformatics analyses. TSK assisted with ex vivo muscle physiology and histology experiments. TS assisted with the ChIP-qPCR experiments. TCL advised on in vivo experimental design and execution and assisted with data analysis and interpretation. EP and DPK wrote the manuscript. All authors participated in scientific discussions, including data analysis, and edited and approved the manuscript.

## Funding support

This work is supported, in part, by NIH funding and is subject to the NIH Public Access Policy. The NIH has been given the right to make the work publicly available in PubMed Central.

- NIH grants R01HL128349 and R35HL177035 (to DPK).
- NIH grant S10-OD025098 (to the Rodent Metabolic Phenotyping Core at the Perelman School of Medicine).

## Supplementary Material

Supplemental data

Unedited blot and gel images

Supporting data values

## Version 1 10/21/2025

In-Press Preview

## Version 2 11/24/2025

Electronic publication
