# Supplementary material for: A role for the transcriptional coregulator RIP140 in the control of muscle endurance fitness
Source: JCI Insight. 2025 Oct 21;10(22):e192376. doi: 10.1172/jci.insight.192376 (PMC12643488; doi:10.1172/jci.insight.192376)
Supplement: Supplemental data [file jciinsight-10-192376-s200.pdf]

Pruzinsky et al, Supplemental Figure 1

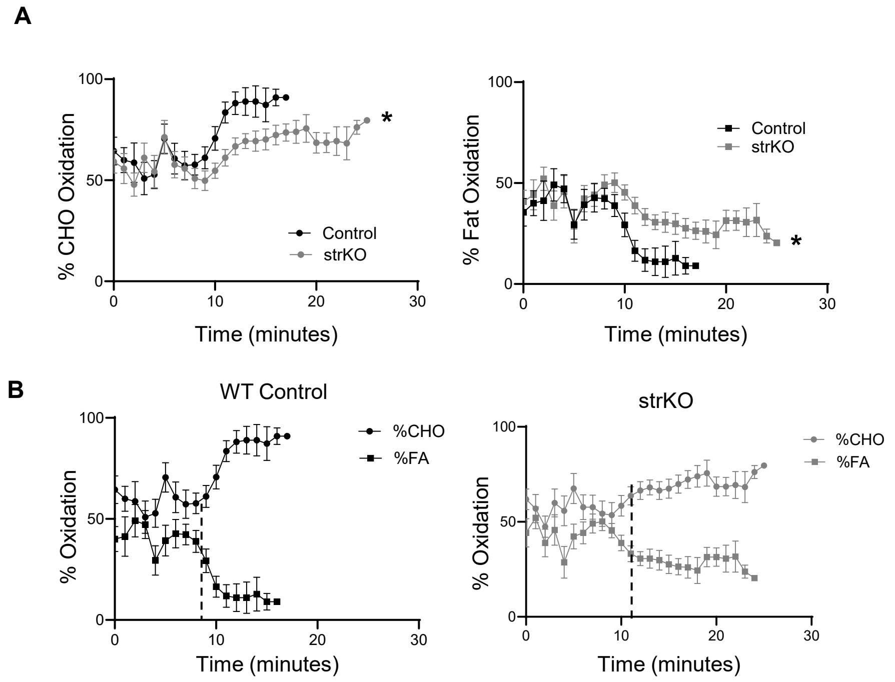

Supplemental Figure 1. str*Nrip1*$^{-/-}$ (strKO) mice display a greater utilization of fatty acids during exercise. (A) Percent carbohydrate (CHO) and fat oxidation calculated from RER measurements derived from the VO_{2max} testing protocol comparing 8-week-old control and strKO mice (n=9 mice per group). **(B)** Crossover oxidation and aerobic threshold (displayed by the dotted line) represented in WT control and strKO mice derived from %CHO and %FA oxidation measurements (n=9 mice per group). Values are the mean ± SEM. *$P < 0.05$ versus control, by 2-tailed, unpaired Student's t test.

Supplemental Figure 2

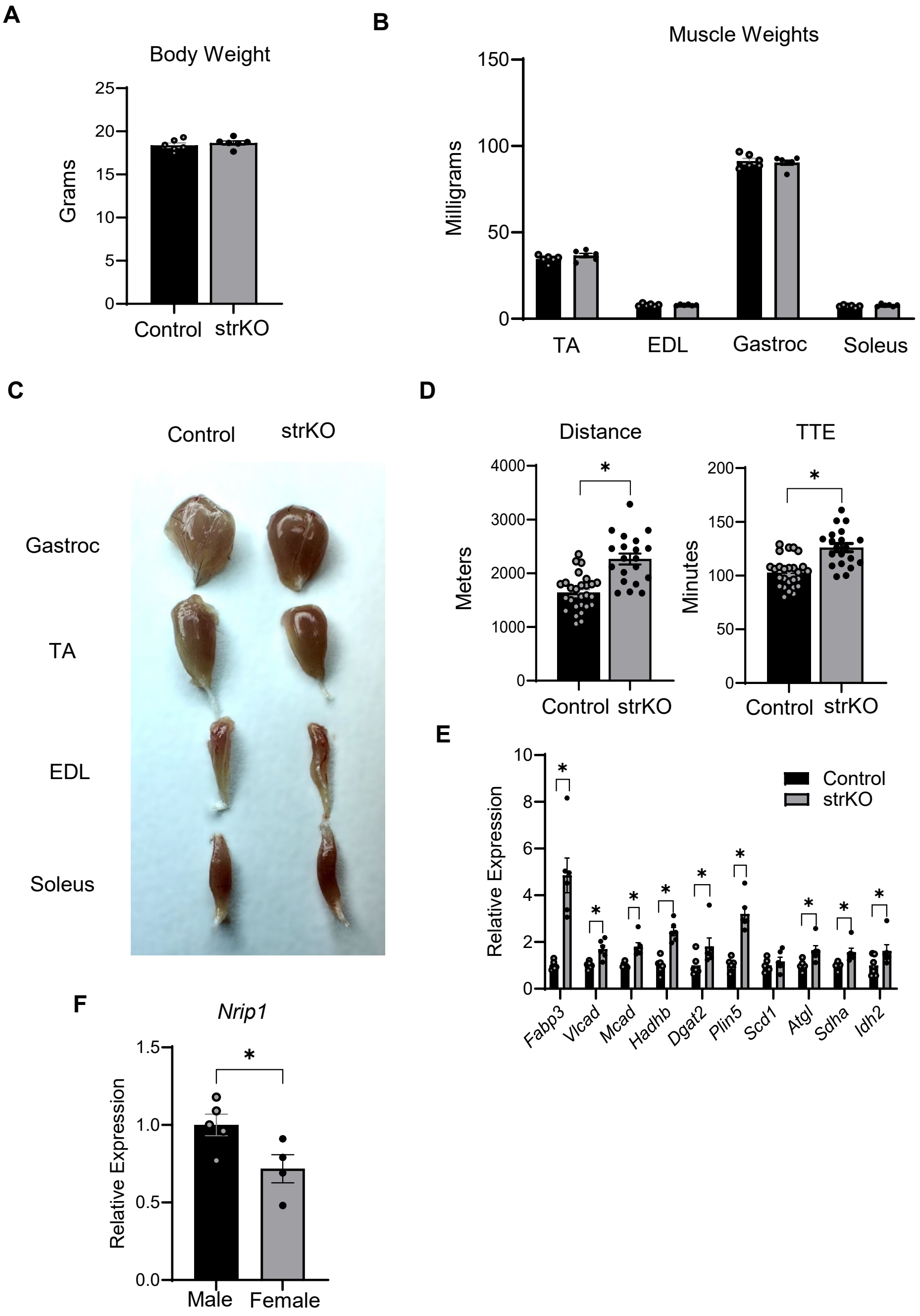

Supplemental Figure 2. Female (str*Nrip1*$^{-/-}$) strKO mice display a similar phenotype to male strKO mice. (A) Body weights of 8-week-old WT control and str*Nrip1*$^{-/-}$ (strKO) female mice (n=6 mice per group). **(B)** Muscle weights (tibialis anterior (TA), extensor digitalis longus (EDL), gastrocnemius (gastroc) and soleus) of 8-week-old WT control and strKO female mice (n=6 mice per group). **(C)** Individual muscle photos as denoted of 8-week-old WT control and strKO female mice. **(D)** Endurance performance results showing distance and time to exhaustion comparing 8-week-old female WT control and strKO mice (n=20-25 per group). **(E)** Gene expression of fatty acid oxidation and lipid droplet remodeling genes measured by qPCR comparing 8-week-old control and strKO female mice (n=5-6 mice per group). **(F)** Nrip1 gene expression measured by qPCR comparing control male and female 8-week-old mice (n=4-5 mice per group). Values are the mean ± SEM. *P < 0.05 by 2-tailed, unpaired Student's t-test.

Supplemental Figure 3

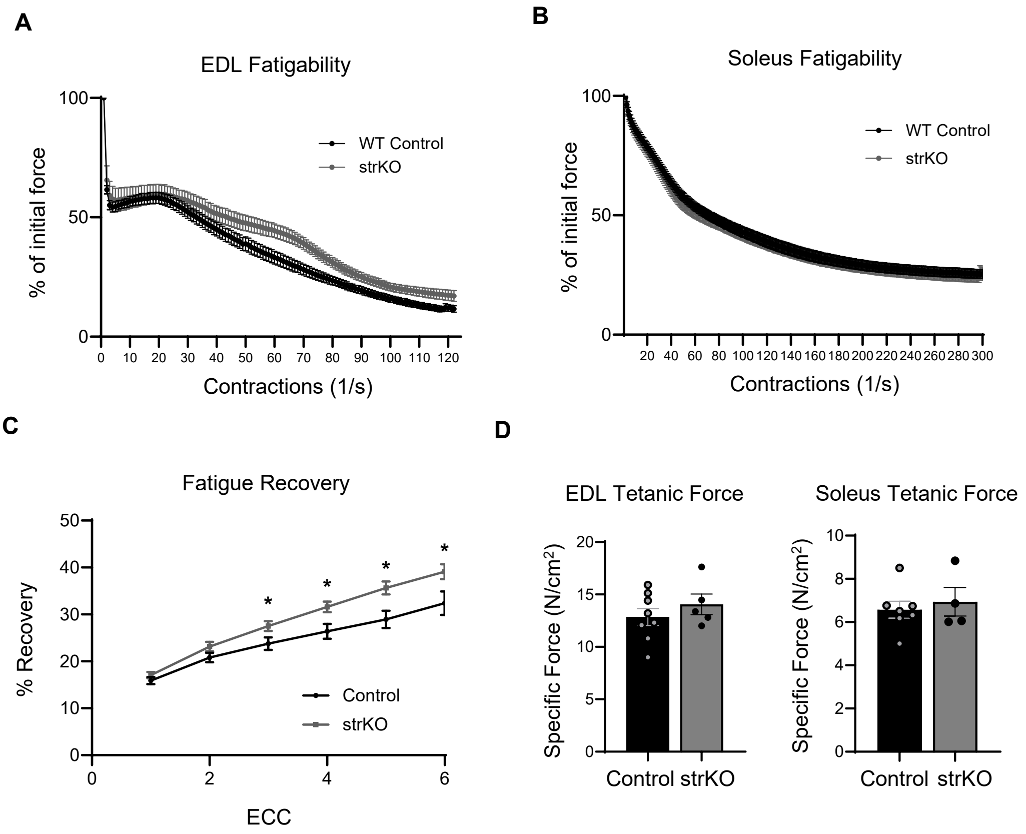

Supplemental Figure 3. Isolated str*Nrip1*$^{-/-}$ skeletal muscle exhibits decreased fatigability. (A) *Ex vivo* fatigability curves comparing 16-week-old male WT control and str*Nrip1*$^{-/-}$ (strKO) extensor digitalis longus (EDL) muscle (n=6-8 mice per group). **(B)** *Ex vivo* fatigability curves comparing 16-week-old male WT control and strKO soleus muscle (n=5-6 mice per group). **(C)** Percent of tetanic force recovery following the *ex vivo* fatigue protocol in male WT control and strKO EDL muscle (n=5-7 mice per group). **(D)** *Ex vivo* tetanic force comparing EDL and soleus from male WT control and strKO muscle (n=4-8 per group). Values are the mean ± SEM. **P* < 0.05 versus control, by 2-tailed, unpaired Student's *t* test.

Supplemental Figure 4

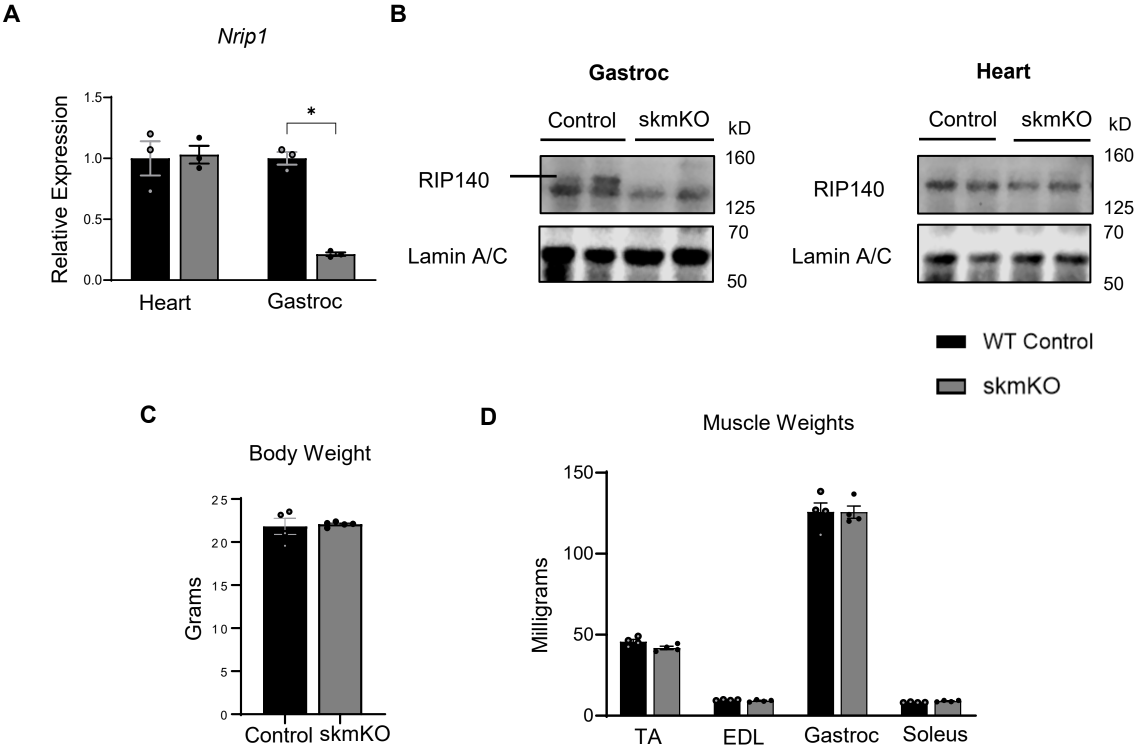

Supplemental Figure 4. Myogenin-Cre RIP140 KO (skm*Nrip1*$^{-/-}$) mouse generation and validation. (A) *Nrip1* expression from heart and gastrocnemius (gastroc) muscle comparing 8-week-old WT control and skm*Nrip1*$^{-/-}$ (skmKO) male mice (n= 3 per group). **(B)** RIP140 western blot of gastrocnemius and heart from WT control and skmKO male mice (n= 5 per group). **(C)** Body weights of 8-week old male control and skmKO mice (n= 4-5 per group). **(D)** Muscle weights from 8-week old control and skmKO male mice (n= 4-5 per group). Values are the mean ± SEM. *P < 0.05 versus control, by 2-tailed, unpaired Student's *t* test.

Supplemental Figure 5

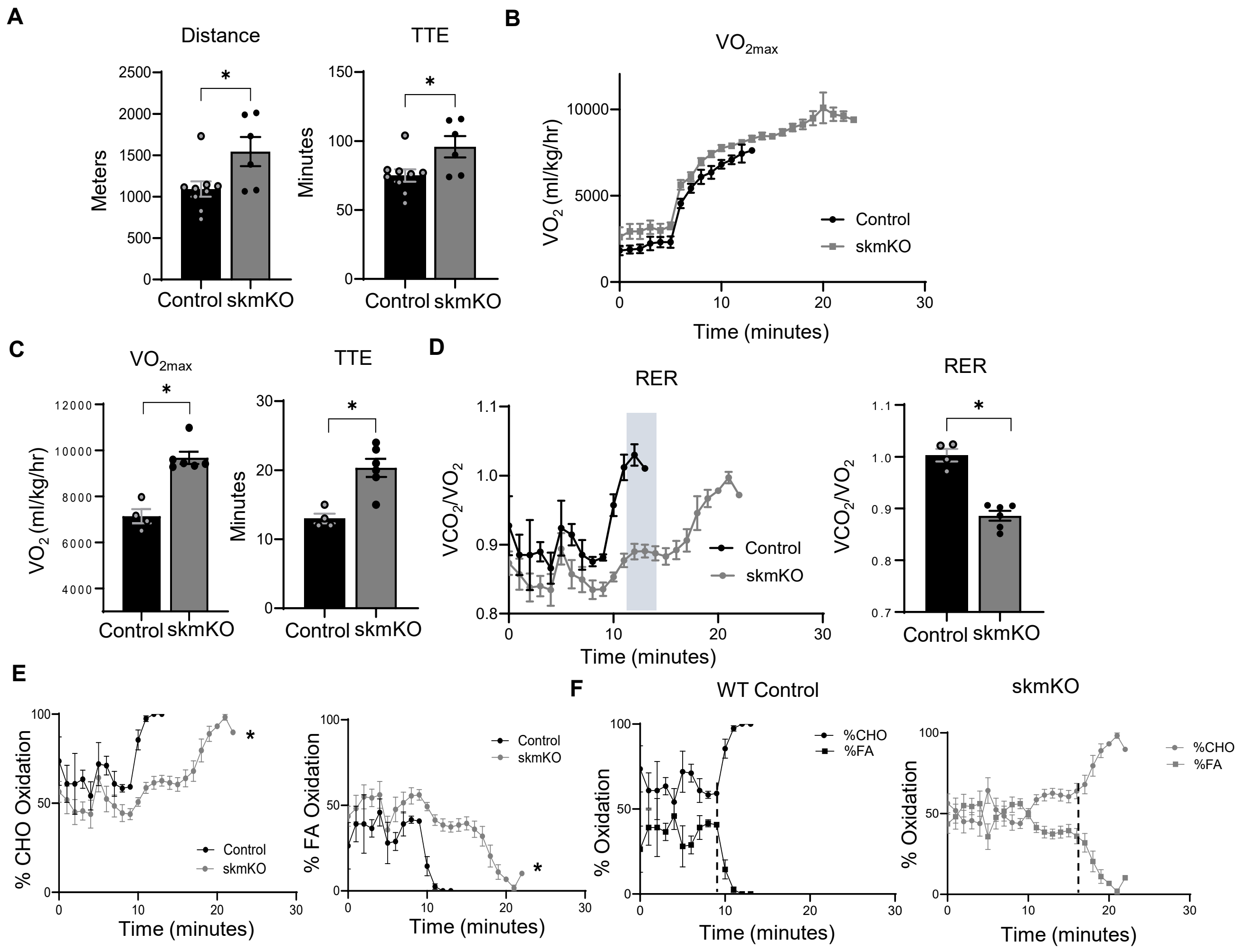

Supplemental Figure 5. skm*Nrip1*$^{-/-}$ (skmKO) mice phenocopy the enhanced endurance phenotype of str*Nrip1*$^{-/-}$ (strKO) mice. (A) Endurance performance results showing distance and time to exhaustion (TTE) comparing 8-week-old WT control and skm*Nrip1*$^{-/-}$ *(*skmKO) male mice (n=6-8 per group). **(B)** VO_2 comparing 8-week-old WT control and skmKO male mice plotted against time during the VO_{2max} exercise protocol (n=4-6 mice per group). **(C)** VO_{2max} and time to exhaustion (TTE) comparing WT control and skmKO mice from the VO_{2max} exercise protocol (n=4-6 per group). **(D)** Respiratory Exchange Ratio (RER) comparing WT control and skmKO male mice throughout the VO_{2max} protocol and plotted at the point of control exhaustion (n=4-6 mice per group). **(E)** Percent carbohydrate (CHO) and fat (FA) oxidation calculated from RER measurements derived from the VO_{2max} testing protocol comparing 8-week-old control and skmKO mice (n=4-6 mice per group). **(F)** Crossover oxidation and aerobic threshold (displayed by the dotted line) represented in WT control and strKO mice derived from %CHO and %FA oxidation measurements (n=4-6 mice per group). Values are the mean ± SEM. *P < 0.05 by 2-tailed, unpaired Student's t-test.

Supplemental Figure 6

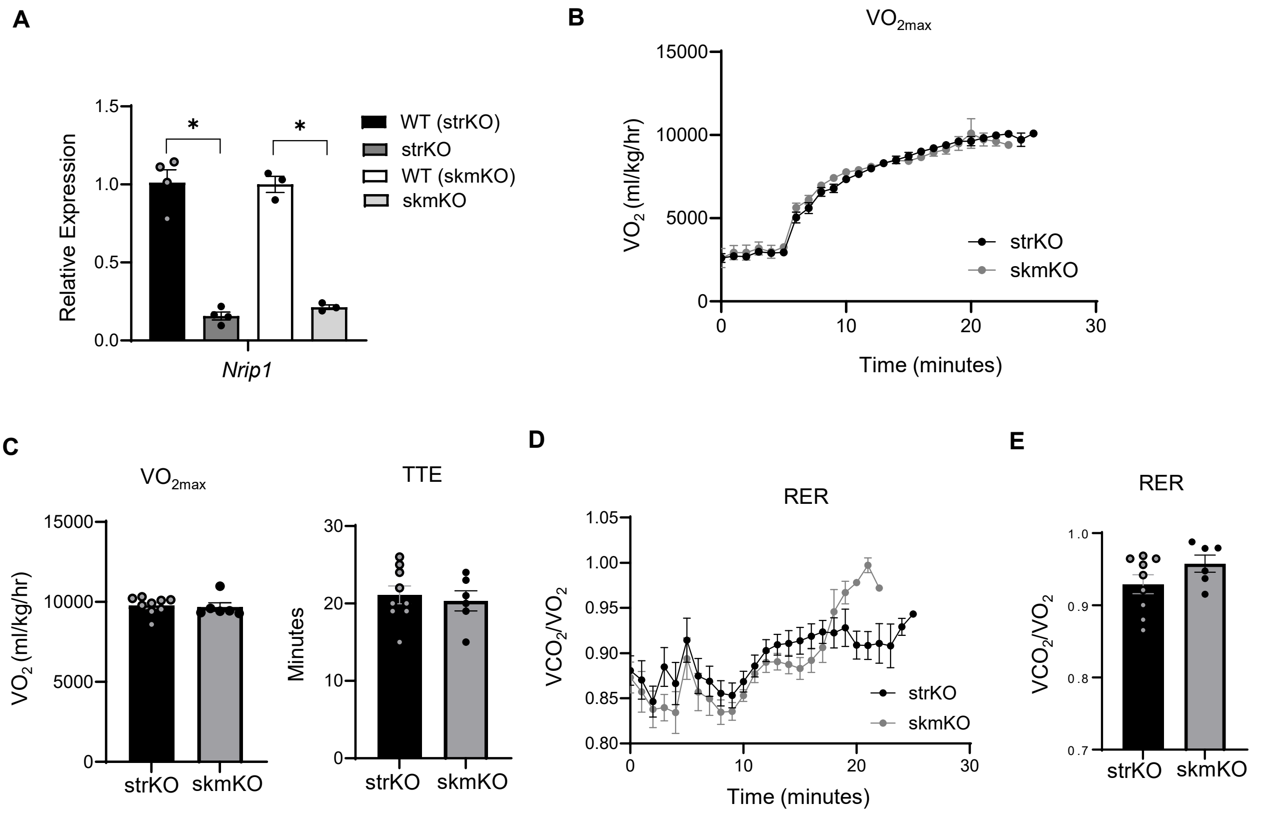

Supplemental Figure 6. strNrip1$^{-/-}$ and skmNrip1$^{-/-}$ display no differences in exercise phenotype. (A) Comparison of *Nrip1* gene expression measured by RT-qPCR comparing 8-week-old strNrip1$^{-/-}$ (WT and strKO) and skmNrip1$^{-/-}$ (WT and skmKO) male mice (n= 3-4 mice per group). **(B)** VO_2 plotted against time of the VO_{2max} exercise protocol comparing strKO and skmKO mice (n= 6-9 mice per group). **(C)** VO_{2max} values and time to exhaustion (TTE) from the VO_{2max} exercise protocol comparing strKO and skmKO mice (n= 6-9 mice per group). **(D)** Respiratory exchange ratio (RER) derived from the VO_{2max} exercise protocol comparing strKO and skmKO mice (n= 6-9 mice per group). **(E)** RER values at exhaustion comparing strKO and skmKO mice (n= 6-9 mice per group). Values are the mean ± SEM. **P* < 0.05 versus control, by 2-tailed, unpaired Student's *t* test.

Supplemental Figure 7

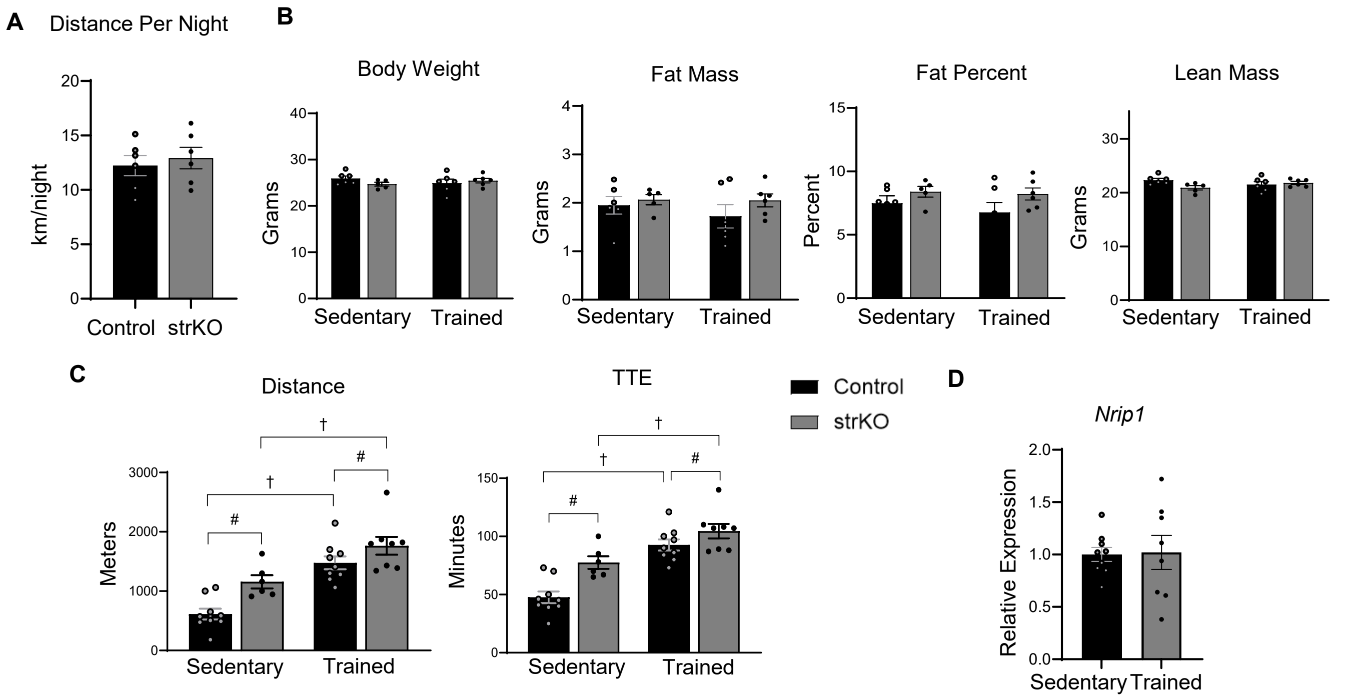

Supplemental Figure 7. Exercise training endpoints. (A) Distance ran per night averaged during the 8-week voluntary wheel training period comparing WT control and str*Nrip1*$^{-/-}$ (strKO) male mice (n= 6 mice per group). **(B)** Body composition including body weight, lean mass, fat mass, and fat percentage among WT control sedentary, strKO sedentary, WT control trained, and strKO trained 16-week-old male mice of the training study (n= 5-6 per group). **(C)** Distance and time to exhaustion from the endurance performance treadmill protocol comparing WT control sedentary, strKO sedentary, WT control trained, and strKO trained 16-week-old male mice of the training study (n= 6-9 per group). **(D)** *Nrip1* gene expression measured by RT-qPCR comparing control sedentary and control trained 16-week-old male mice (n= 8-9 per group). Values are the mean ± SEM. #P < 0.05 comparing genotypes and †P< 0.05 comparing training by 2-way ANOVA displaying main effects or 2-tailed, unpaired Student's *t* test.

Supplemental Figure 8

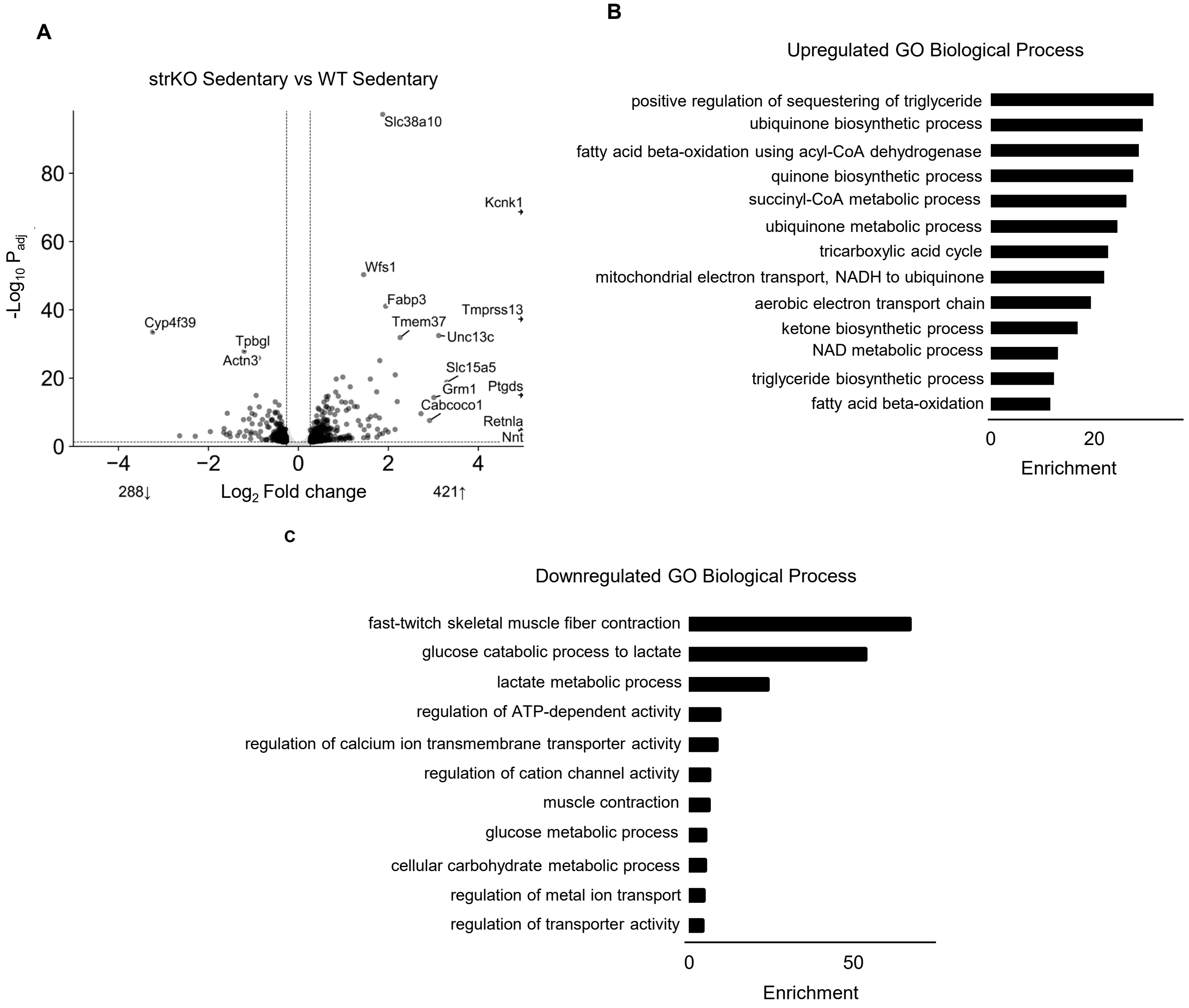

Supplemental Figure 8. RIP140 regulates an array of pathways/processes known to be involved in muscle endurance. (A) Volcano plot of differentially expressed genes from RNA-sequencing comparing 16-week-old male str*Nrip1*$^{-/-}$ (strKO) and WT control sedentary extensor digitalis longus (EDL) muscle (n=3 per group). Fold change (FC) >1.2; FDR <0.05. **(B)** Top 20 upregulated biological process pathways in strKO sedentary EDL compared to WT control sedentary EDL. **(C)** Top 20 downregulated biological process pathways in strKO sedentary EDL compared to control sedentary EDL.

Supplemental Figure 9

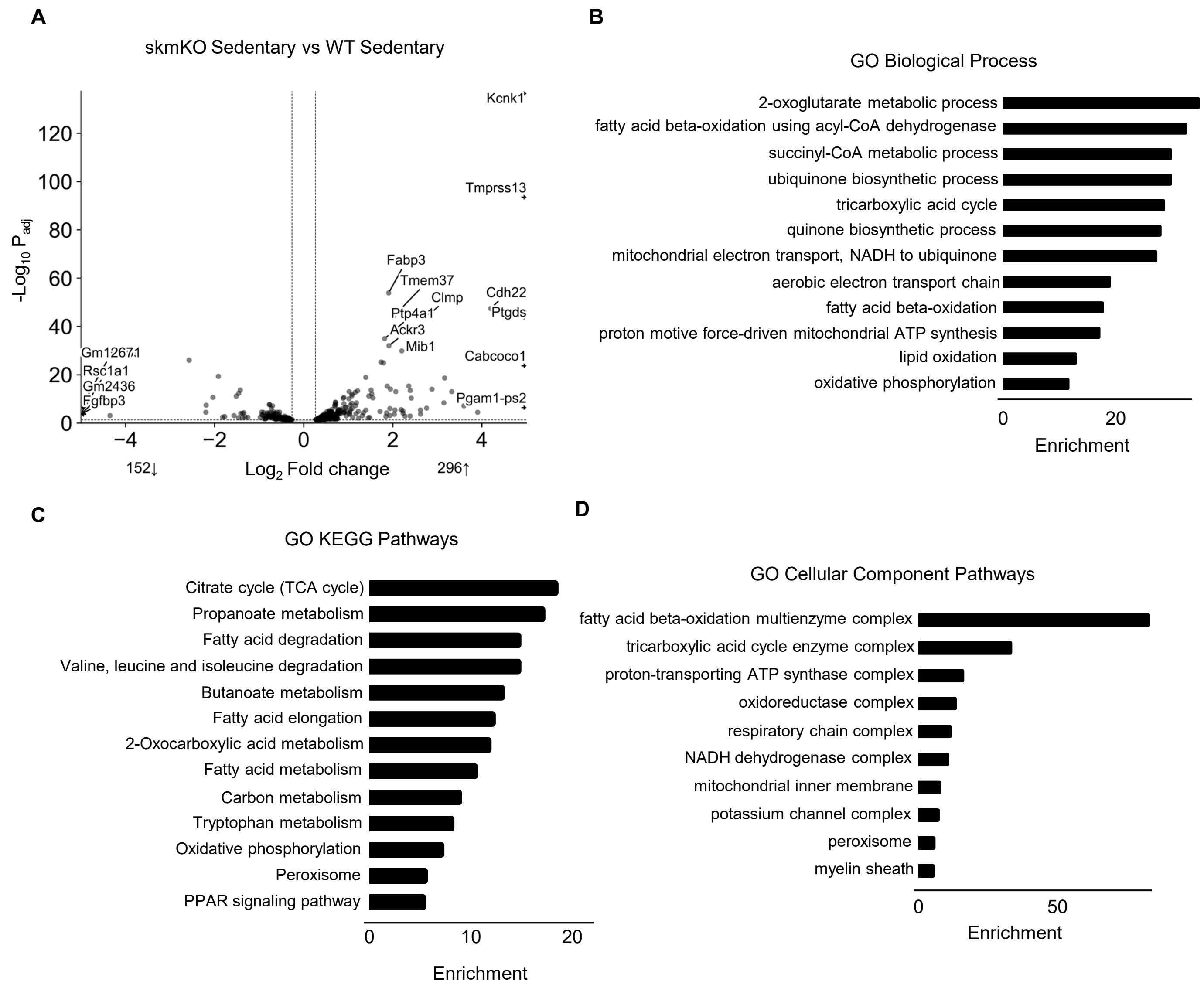

Supplemental Figure 9. Transcriptome of skm*Nrip1*$^{-/-}$ (skmKO) muscle recapitulates str*Nrip1*$^{-/-}$ (strKO) muscle transcriptome. (A) Volcano plot of differentially expressed genes from RNA-sequencing comparing 8-week-old male skmKO and WT control sedentary tibialis anterior (TA) muscle (n= 3 per group). Fold change (FC) >1.2; FDR <0.05. **(B)** Top upregulated enriched biological process pathways in skmKO sedentary TA compared to control sedentary TA. **(C)** Top upregulated KEGG pathways in skmKO sedentary TA compared to WT control sedentary TA. **(D)** Top upregulated enriched cellular compartment pathways in skmKO sedentary TA compared to control sedentary TA.

Supplemental Figure 10

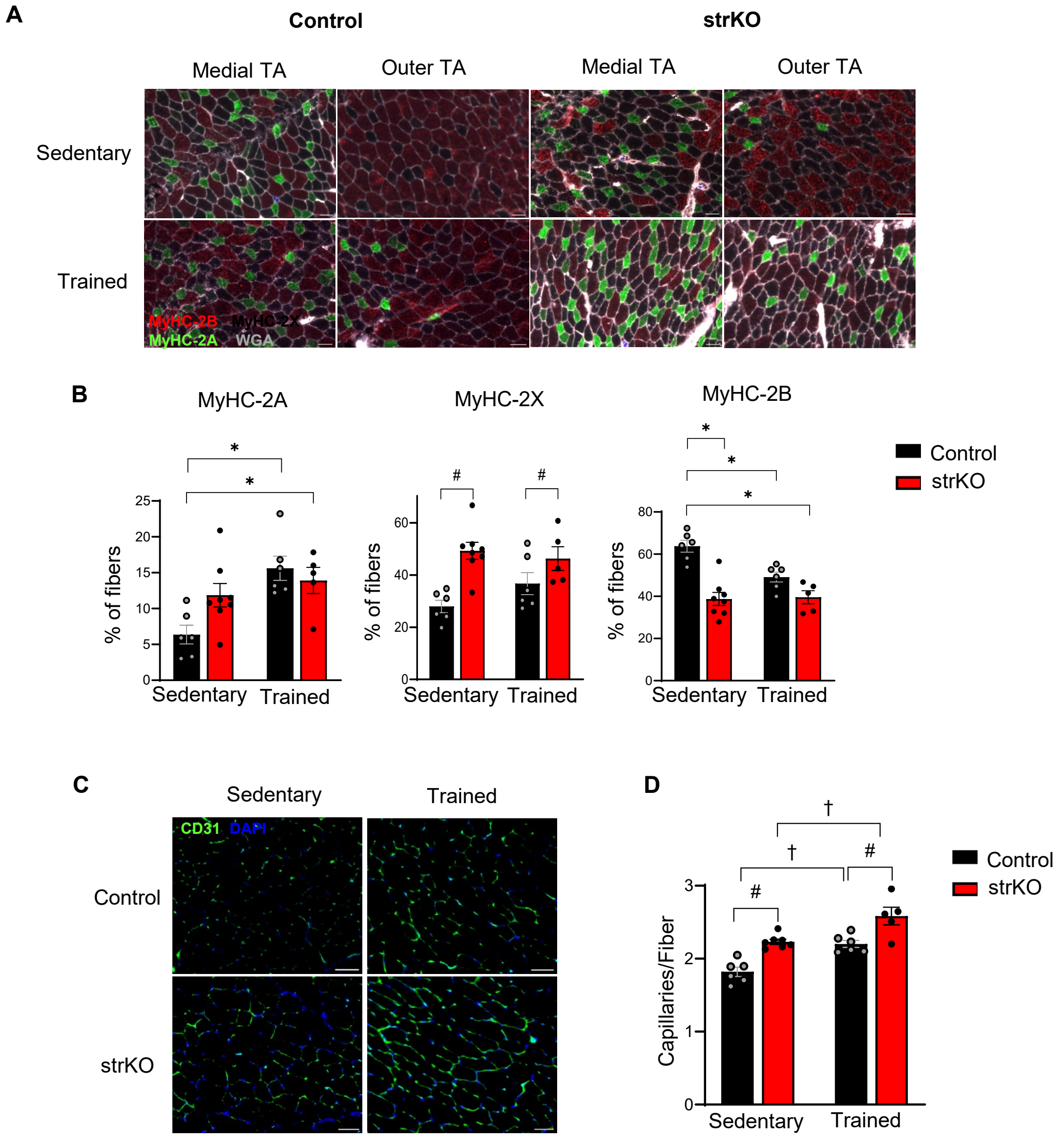

Supplemental Figure 10. str*Nrip1*$^{-/-}$ (strKO) mice display a shift in fiber type and increased angiogenesis. (A) Fiber type staining of tibialis anterior (TA) muscle displaying outer and medial areas from 16-week-old male strKO sedentary, control sedentary, strKO trained, and WT control trained mice (n=5-8 mice per group). Scale bar: 50 µm. **(B)** Quantification of myosin expression (MyHC-2A, MyHC-2X, and MyHC-2B) from TA fiber type images of male strKO sedentary, control sedentary, strKO trained, and WT control trained mice. **(C)** CD31 staining (green) in TA muscle from 16-week-old male strKO sedentary, control sedentary, strKO trained, and WT control trained mice (n=5-7 mice per group). Scale bar: 50 µm. **(D)** Quantification of capillaries per muscle fiber from CD31 TA immunofluorescence images from strKO sedentary, control sedentary, strKO trained, and WT control trained mice (n=5-6 mice per group). Values are the mean ± SEM. #P < 0.05 comparing genotypes and †P< 0.05 comparing training by 2-way ANOVA displaying main effects or *P<0.05 by Tukey's multiple-comparison test.

Supplemental Table 1. qPCR primers.

Gene	Forward	Reverse
Acsl1	CGCCCATATGTTTGAGACCG	GTCGTCCATAAGCAGCCTGA
Bdnf	TGGCCCTGCGGAGGCTAAGT	AGGGTGCTTCCGAGCCTTCCT
Cd36	TGGAGTGGTGATGTTTGTTGC	AGCCAGTGTATATGTAGGCTCA
Chrna2	AAAGTCACGCTTGCAGACTC	GATGTTGCCAAACTCAGCCG
Dgat2	GCATTTGACTGGAACACGCC	CTGGTGGTCAGCAGGTTGTG
Dgat1	GCGACGGCTACTGGGATCTG	TGCATTACTCAGGATCAGCATCA
Fabp3	AAGTGGAACGGGCAGGAGA	GAGGAGCGGGCGGTCAG
Gk	CCGTTACTCCACATGGAAGAAAGCTGTG	GCATCTTGGAAATCCGTGAGGTGG
Musk	TGAGAACTGCCCCTTGGAACT	GGGTCTATCAGCAGGCAGCTT
Nrip1	GAGGCCCGGAGAATCTGAAG	TTTCGTTGCTCACCAAACGC
Plin2	GACCTTGTGTCCTCCGCTTAT	CAACCGCAATTTGTGGCTC
Plin5	GGATCACTTCCTGCCCATGAC	ATCCTCCACCGAACCCACTTC
Pnpla2	CAACGCCACTCACATCTACGG	TGAAGGAGGGATGCAGAGGAC
Scd1	GCAAGGTAATGTGGCTTTGGCTGA	TTAGCACTTGCCCATGTCTCTGGT
Wnt16	AGTAGCGGCACCAAGGAGAC	GAAACTTTCTGCTGAACCACATGC
Unc13c	TCTGACACCAAGACAATGTGC	AGCAATATTTTCATGCAGGAGG

Supplemental Table 2. ChIP-qPCR primers.

Gene	Forward	Reverse
Dgat2	CGCGCGCACTTTATAACCTC	GGGAGTCTCACCCCCTACAA
HNFα	TGGCCGTGAGCATCCTCTGCC	GCGTGGGTTGCGTTTGCCTGC
Wnt16	AAGAAGCTCCTGGGCGAAAG	GCAAGAAGGGGGTGTTCTGA
