# Supplementary material for: A role for the transcriptional coregulator RIP140 in the control of muscle endurance fitness
Source: JCI Insight. 2025 Oct 21;10(22):e192376. doi: 10.1172/jci.insight.192376 (PMC12643488; doi:10.1172/jci.insight.192376)

Full Unedited blot for Figure 7A

TA blot (MUSK)

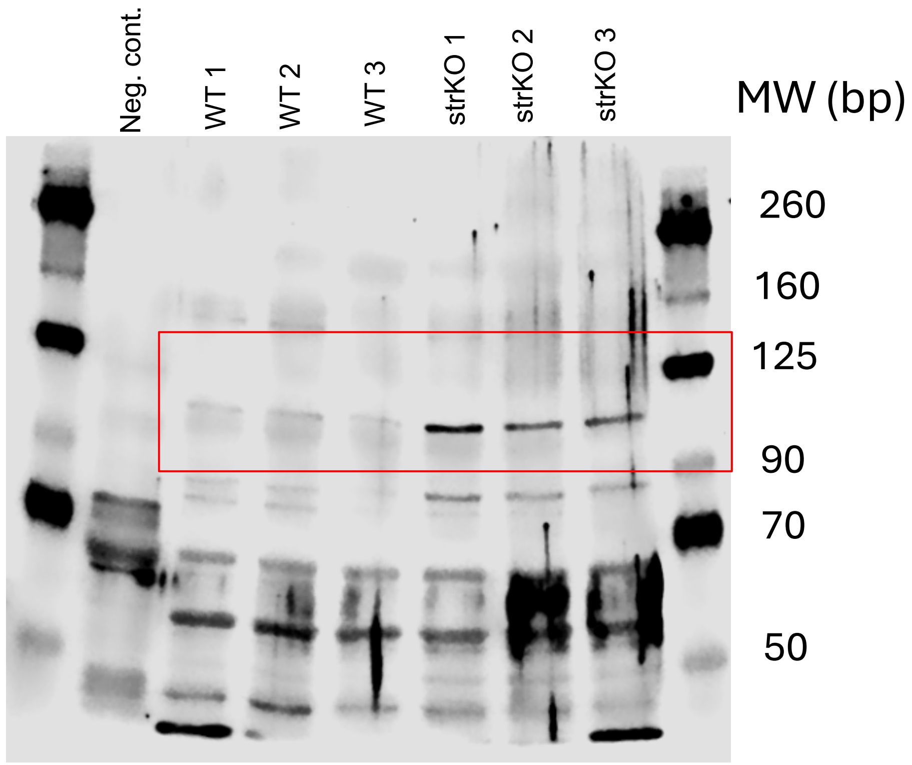

TA blot (α-Tubulin)

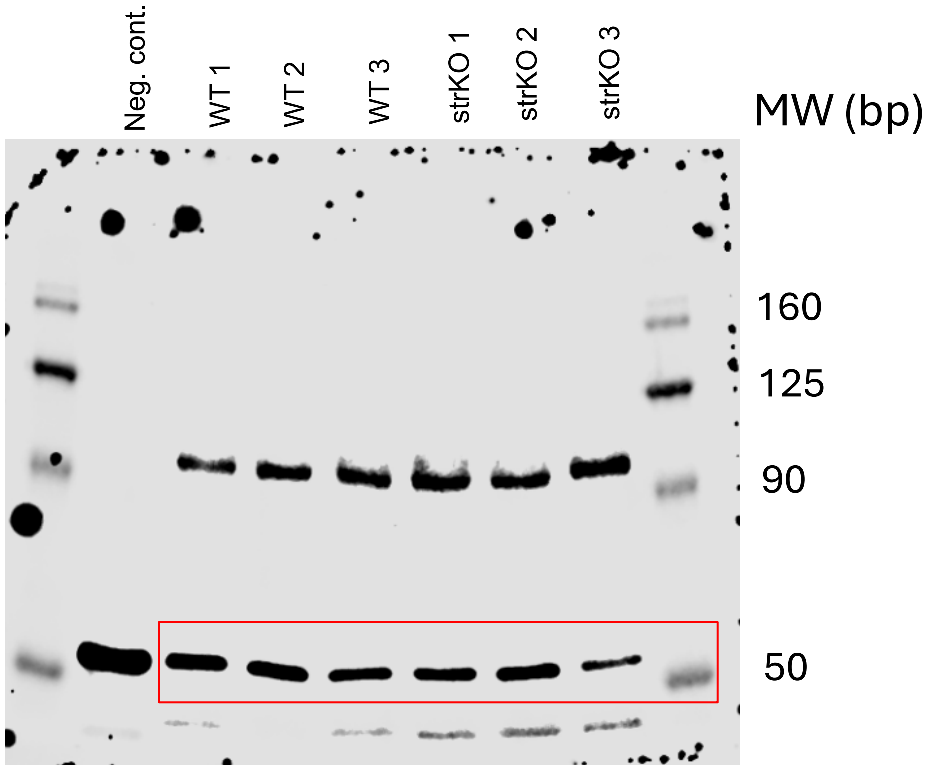

Unedited blot for Supplemental Figure 4B

Gastroc blot (RIP140)

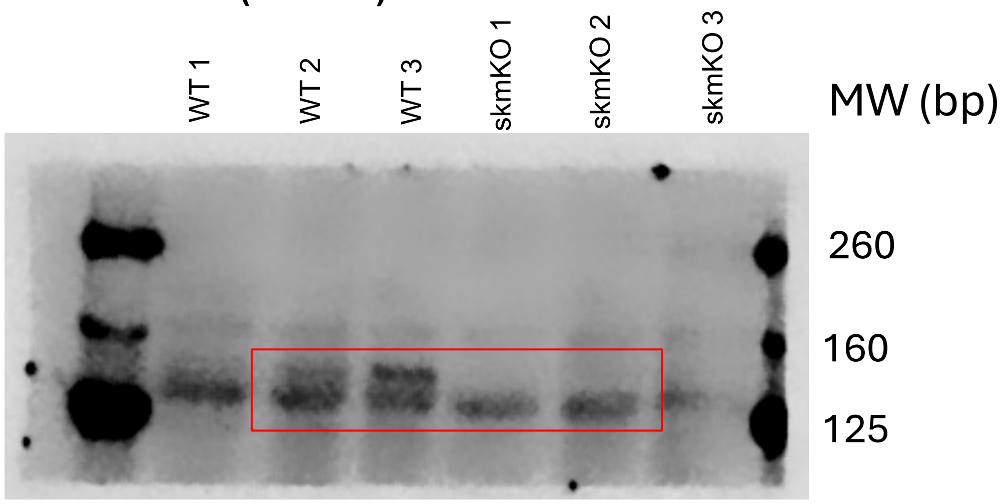

Gastroc blot (Lamin A/C)

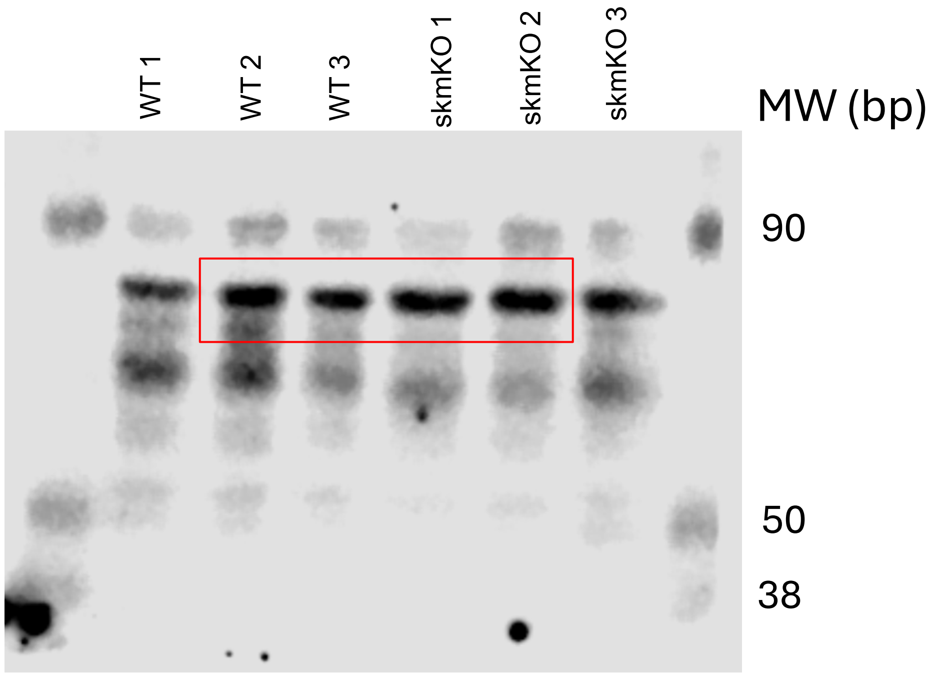

Unedited blot for Supplemental Figure 4B

Heart blot (RIP140)

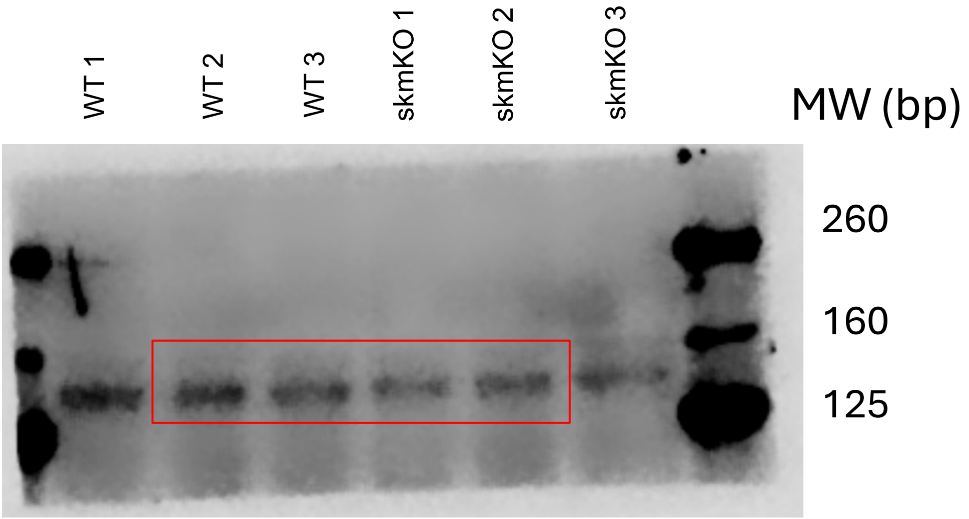

Heart blot (Lamin A/C)

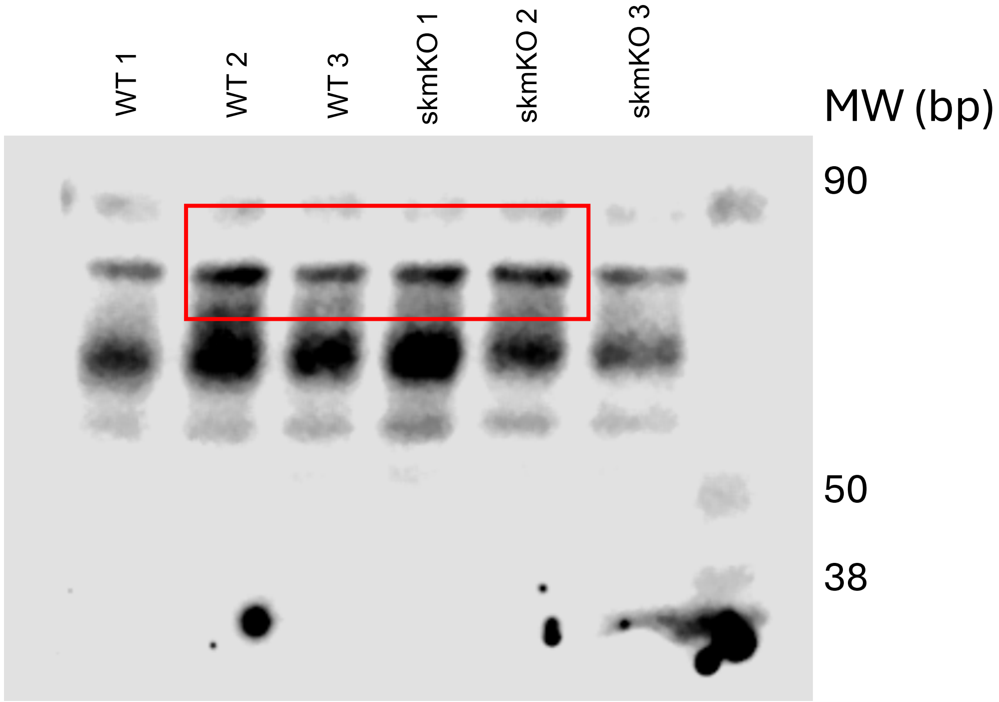

Supplement: Unedited blot and gel images [file jciinsight-10-192376-s201.pdf]
